# Inhibition of miR-200b-3p confers broad-spectrum resistance to viral infection by targeting TBK1

**DOI:** 10.1128/mbio.00867-23

**Published:** 2023-05-24

**Authors:** An Fang, Yueming Yuan, Baokuen Sui, Zhihui Wang, Yuan Zhang, Ming Zhou, Huanchun Chen, Zhen F. Fu, Ling Zhao

**Affiliations:** 1 State Key Laboratory of Agricultural Microbiology, Huazhong Agricultural University, Wuhan, China; 2 Key Laboratory of Preventive Veterinary Medicine of Hubei Province, College of Veterinary Medicine, Huazhong Agricultural University, Wuhan, China; 3 Hubei Hongshan Laboratory, Wuhan, China; Virginia Polytechnic Institute and State University, Blacksburg, Virginia, USA; University of Georgia College of Veterinary Medicine, Athens, Georgia, USA

**Keywords:** innate immunity, interferon, TBK1, microRNA-200b-3p, broad-spectrum, viral infection, influenza virus

## Abstract

The host innate immune system’s defense against viral infections depends heavily on type I interferon (IFN-I) production. Research into the mechanisms of virus-host interactions is essential for developing novel antiviral therapies. In this study, we compared the effect of the five members of the microRNA-200 (miR-200) family on IFN-I production during viral infection and found that miR-200b-3p displayed the most pronounced regulatory effect. During viral infection, we discovered that the transcriptional level of microRNA-200b-3p (miR-200b-3p) increased with the infection of influenza virus (IAV) and vesicular stomatitis virus (VSV), and miR-200b-3p production was modulated by the activation of the ERK and p38 pathways. We identified cAMP response element binding protein (CREB) as a novel transcription factor that binds to the miR-200b-3p promoter. MiR-200b-3p reduces NF-κB and IRF3-mediated IFN-I production by targeting the 3′ untranslated region (3′ UTR) of TBK1 mRNA. Applying miR-200b-3p inhibitor enhances IFN-I production in IAV and VSV-infected mouse models, thus inhibiting viral replication and improving mouse survival ratio. Importantly, in addition to IAV and VSV, miR-200b-3p inhibitors exhibited potent antiviral effects against multiple pathogenic viruses threatening human health worldwide. Overall, our study suggests that miR-200b-3p might be a potential therapeutic target for broad-spectrum antiviral therapy.

**IMPORTANCE:**

The innate immune response mediated by type I interferon (IFN-I) is essential for controlling viral replication. MicroRNAs (miRNAs) have been found to regulate the IFN signaling pathway. In this study, we describe a novel function of miRNA-200b-3p in negatively regulating IFN-I production during viral infection. miRNA-200b-3p was upregulated by the MAPK pathway activated by IAV and VSV infection. The binding of miRNA-200b-3p to the 3′ UTR of TBK1 mRNA reduced IFN-I activation mediated by IRF3 and NF-κB. Application of miR-200b-3p inhibitors exhibited potent antiviral effects against multiple RNA and DNA viruses. These results provide fresh insight into understanding the impact of miRNAs on host-virus interactions and reveal a potential therapeutic target for common antiviral intervention.

## INTRODUCTION

Host innate immunity serves as the first line of defense against viral infections. Upstream of innate immunity are several types of pathogen recognition receptors (PRRs), such as retinoic acid-inducible gene (RIG)-I-like receptor (RLR) and cyclic GMP-AMP synthase (cGAS), which can be activated by the binding of viral RNA or DNA. PRRs recruit various downstream adaptor proteins, such as mitochondrial antiviral-signaling protein (MAVS), or stimulator of interferon genes, which subsequently transduce signals through TANK-binding kinase 1 (TBK1), thereby activating IFN regulatory factor 3 (IRF3) and nuclear factor-κB (NF-kB) to induce IFN-I production (1, 2). Binding of IFN-I to its receptor leads to the activation of the JAK-STAT signaling cascade, which induces the expression of more than 300 interferon-stimulated genes (ISGs) that directly limit viral replication and promote protective responses against viral infection (3 - 5).

TBK1 is a ubiquitous serine/threonine kinase protein that plays a critical role in several signaling pathways. TBK1 has been proposed as a noncanonical IκB kinase (IKK). Canonical IKKs (IKKα/β) are important for the broad NF-κB signaling pathways that control cell survival, growth, and cancer progression (6, 7). TBK1 activates the NF-κB and IRF3 pathways in the context of infection and autoimmune disease (8). TBK1 activates the NF-κB signaling pathway by phosphorylating several key members of the pathway (including RelA, cRel, and IKBα) (9). In addition, activated TBK1 directly phosphorylates several IRF3/7 Ser and Thr residues. Phosphorylation of Ser386 and Ser396 on IRF3 is critical for IRF3 activation, whereas phosphorylation of Ser 477 and 479 on IRF7 is required for IRF7 activation (10). Phosphorylated IRFs form homo- and/or heterodimers that translocate to the nucleus and bind IFN-stimulated response elements (ISREs) in target gene promoters, leading to type I IFN expression (2).

miRNAs are small noncoding RNAs of 18–22 nucleotides in length. They are mostly encoded by gene introns and sometimes by dedicated genes (11). miRNAs regulate the expression of specific target proteins by inhibiting translation or degrading the corresponding mRNAs (12). MiRNAs are involved in most of the major functions of cells, such as growth, proliferation, differentiation, signal transduction, apoptosis, metabolism, and aging (13, 14). Recent studies have identified an important contribution of miRNAs to the regulation of the innate immune response (15). There is evidence that some miRNAs, such as miR-146, miR-9, miR-147, miR-21, and miR-155, can negatively regulate the activation of inflammatory pathways. Among these miRNAs, miR-146 directly inhibits several signaling molecules downstream of TLRs, including IL-1R related kinase 1 (IRAK1), IRAK2, and TNFR related factor 6 (16 - 20). Other studies have shown that miRNAs can modulate the IFN signaling pathway. miR-466l directly binds to the IFN-α 3′ UTR and reduces IFN-α expression during VSV infection (21).

The miR-200 family includes five members, miR-200a-3p, miR-200b-3p, miR-200c-3p, miR-141-3p, and miR-429 and has been extensively studied for its important role in cancer (22 - 27). Recent studies have reported significant changes in miR-200 family expression upon viral infection, and in particular, microRNA-200c inhibited IAV replication by upregulating IFN-I production (28, 29). In this study, we compare the effect of miR-200 family on IFN-I production and find that miR-200b-3p suppresses IFN-I activation by targeting TBK1. Our data highlight the broad-spectrum antiviral effect of miR-200b-3p inhibitor, which provides a clue for the development of a novel therapeutic strategy against viral infection.

## MATERIALS AND METHODS

### Cells, viruses, antibodies, inhibitors, and mice

Dulbecco’s modified Eagle’s medium (DMEM) (Thermo Fisher Scientific) or RPMI 1640 medium (HyClone, SH30809.01) supplemented with 10% (vol/vol) fetal bovine sera (FBS) (Thermo Fisher Scientific, Waltham, MA, USA) and 1% antibiotics (penicillin and streptomycin) (Beyotime, Shanghai, China) were used to culture HEK-293T (human embryonic kidney, 293T), N2a (mouse neuroblastoma), BHK-21 (baby hamster kidney-21), BSR (a clone of BHK-21), Vero (African green monkey kidney), and MDCK (Madin-Darby canine kidney) cells. IAV PR8/H1N1 strain is donated by Dr. Hongbo Zhou (Huazhong Agricultural University, Wuhan, Hubei, China) and propagated in chicken embryos. VSV was propagated in N2a cells and stored in our lab. RABV (CVS-B2c strain, originated from CVS-24 virus by passaging in BHK-21 cells) is propagated in N2a cells and stored in our lab (30). JEV (P3 strain) was donated by Dr. Shengbo Cao (Huazhong Agricultural University) and propagated in suckling mouse brains. HSV-1-GFP was donated by Dr. Bo Zhang (Wuhan Institute of Virology, Wuhan, Hubei, China) and propagated in Vero cells. Sendai virus (SeV, Cantell strain, Charles River Laboratories) was propagated in chicken embryos and used at a final concentration of 100 hemagglutinin units per mL.

The monoclonal antibody (mAb) against IAV NP protein (#IT-003-023M1) was purchased from Immune Technology. The mAb against RABV N protein was prepared by our lab. The mAb against VSV G protein (#81454) was purchased from CST. The mAb against Sendai virus HN protein (#14-6494-82) was purchased from Thermo Fisher Scientific. The polyclonal antibody (pAb) against p65, p-p65, JNK, p-JNK, ERK, p-ERK, p38, p-p38, CREB, Lamin B1, RIG-I, MAVS, TBK1, IRF3, p-IRF3, IRF7, p-IRF7, IKBα, p-IKBα, STAT1, p-STAT1, ISG15, and GFP were purchased from ABclonal. The mAb against Flag (#M185-3L) and β-Actin (#M177-3) was purchased from MBL.

The JNK inhibitor (no. GC13841), the NF-kB inhibitor (no. GC11751), the p38 inhibitor (no. GC18602), the ERK inhibitor (no. GC43624), Asperuloside (no. GC35411), and the CREB inhibitor (no. GC32689) were all purchased from Glpbio (Montclair, CA). All the experiments involving mice were performed following the recommendations in the Guide for the Care and Use of Laboratory Animals of the Ministry of Science and Technology of China and were approved by the Scientific Ethics Committee of Huazhong Agricultural University (permit number: HZAUMO-2017-056).

### MicroRNA mimics and inhibitors

MicroRNA mimics, inhibitors, and control oligonucleotides were commercially synthesized by GenePharma (Shanghai, China). MicroRNA mimics are small, chemically modified double-stranded RNA molecules designed to specifically mimic endogenous miRNA molecules. The sequences of microRNA mimics are as follows: NC mimics: 5′-UUCUCCGAACGUGUCACGUTT-3′; hsa-miR-200b-3p mimics: 5′-UAAUACUGCCUGGUAAUGAUGA-3′; hsa-miR-200c-3p mimics: 5′- 5′-UAAUACUGCCGGGUAAUGAUGGA-3′; hsa-miR-200a-4p: 5′-UAACACUGU CUGGUAACGAUGU-3′; hsa-miR-429: 5′-UAAUACUGUCUGGUAAAACCGU-3′; and hsa-miR-141-3p: 5′-UAACACUGUCUGGUAAAGAUGG-3′.

Has-miR-200b-3p inhibitors are single-stranded oligonucleotides, which had base-pairing complementarity to miR-200b-3p to block miR-200b-3p function (31). The sequences of inhibitors are as follows: NC inhibitors: 5′-CAGUACUUUUGUGUAGUACAA-3′ and miR-200b-3p inhibitors: 5′-UCAUCAUU ACCAGGCAGUAUUA-3′.

### Viral infection

Cells (293T, N2a, BSR, BHK-21, MDCK, and Vero) were infected with IAV, VSV, RABV, JEV, or HSV-1 at a multiplicity of infection (MOI) of 0.001, 0.01, and 0.1. After 1 h at 37°C, the supernatant was discarded and cells were washed three times with PBS and then cultured in DMEM or RPMI1640 supplemented with 2% (vol/vol) FBS (Gibco) and 1% antibiotics (penicillin and streptomycin, Beyotime) at 34°C in a humidified 5% CO_2_ atmosphere.

### Virus titration

For IAV titration, viral titers of virus stocks and cell culture supernatants were determined by end-point titration in MDCK cells. Tenfold serial dilutions of each sample were inoculated into MDCK cells. Two days after inoculation, supernatant from the inoculated cells was collected and tested for the ability to agglutinate chicken erythrocytes as an indicator of viral replication. Infectious viral titers are reported as log_10_ TCID_50_/mL and were calculated from three replicates by using the method of Reed-Muench (32).

For RABV and VSV titration, N2a cells were infected with serial dilutions of the viruses. After 1 h incubation at 37°C, the cell supernatant was discarded and washed once with PBS, and then overlaid with DMEM containing 1% low melting point agarose (VWR, 2787C340). After incubation at 34°C for 72 h, 293T cells were stained with FITC-conjugated antiRABV N antibody (Fujirebio Diagnostics, Malvern, PA). Then, the fluorescent foci were counted under a fluorescence microscope. For VSV titration, the plaques were counted at 48 h postinfection.

For HSV-1 titration, Vero cells were seeded in 12-well plates and infected with serial dilutions of the viruses. After 1 h incubation at 37°C, the cell supernatant was discarded and washed once with PBS and then overlaid with DMEM containing 1% low melting point agarose. After incubation at 34°C for 48 h, the agarose was removed and then fixed and stained with a solution of 0.1% crystal violet and 10% formalin in PBS under UV light. After staining for 4 h, the plates were washed with water, and the plaques were counted.

For JEV titration, BHK-21 cells were seeded in 12-well plates and infected with serial dilutions of the viruses. After 1 h incubation at 37°C, the cell supernatant was discarded and washed once with PBS, and then overlaid with DMEM containing 1% low melting point agarose. After incubation at 34°C for 48 h, the agarose was removed and then fixed and stained with a solution of 0.1% crystal violet and 10% formalin in PBS under UV light. After staining for 4 h, the plates were washed with water, and the plaques were counted.

### RNA isolation and quantitative real-time PCR (qPCR)

Total RNA was isolated from cells and tissues by using TRIzol reagent (Invitrogen). The genomic DNA was eliminated with TURBO DNA-free Kit (Invitrogen, AM1907) as per the manufacturer’s instructions. RNA quality was assessed by using NanoDrop 2,000 (Thermo Scientific). The cDNAs were synthesized by ReverTra Ace qPCR RT Master Mix (Toyobo, FSQ-201) or First-Strand cDNA Synthesis Kit (Vazyme, R211-01). qPCR was performed using SYBR Green Supermix (Bio-Rad, 172-5124). Primers for qPCR are listed in Table 1

**TABLE 1:** Primers used for qPCR

Primers	Sequence 5′-3′
IFN-β-F	AGATGTCCTCAACTGCTCTC
IFN-β-R	AGATTCACTACCAGTCCCAG
Mx1-F	CAACTGGAATCCTCCTGGAA
Mx1-R	GGCTCTCCTCAGAGGTATCA
OAS1a-F	CCAAGGTGGTGAAGGGTGG
OAS1a-R	ACCACCAGGTCAGCGTCTGA
IFIT3-F	CCTACATAAAGCACCTAGATGGC
IFIT3-R	ATGTGATAGTAGATCCAGGCGT
ISG15- *F*	GGTGTCCGTGACTAACTCCAT
ISG15- R	CTGTACCACTAGCATCACTGTG
TBK1-Hsa-F	TGGGTGGAATGAATCATCTACGA
TBK1-Hsa-R	GCTGCACCAAAATCTGTGAGT
GAPDH-Has-F	GGAGCGAGATCCCTCCAAAAT
GAPDH-Has-R	GGCTGTTGTCATACTTCTCATGG
GAPDH-Mmu -F	AGGTCGGTGTGAACGGATTTG
GAPDH-Mmu -R	TGTAGACCATGTAGTTGAGGTCA
pre-miR-200b-3p-F	CCAGCTCGGGCAGCCGTG
pre-miR-200b-3p-R	CGTGCAGGGCTCCGCCGT
pri-miR-200b-3p-F	CTTCCCAGCGAGTCCCATGC
pri-miR-200b-3p-R	CTGTGTGGGAGGGGAGTGTG
U6-qPCR *F*	CTCGCTTCGGCAGCACA
U6-qPCR R	AACGCTTCACGAATTTGCGT

The method for stem-loop real-time quantification of miRNA was described previously (33), and the schematic diagram is shown in Fig. S10. For miRNA quantification, total RNA from cells was purified using the MiPure Cell/Tissue miRNA Kit (Vazyme, RC201) to retain small RNA according to the manufacturer’s protocol. The cDNAs were synthesized by miRNA 1st Strand cDNA Synthesis Kit (Vazyme, MR101-01). Stem-loop qPCR was performed using ChamQ Geno-SNP Probe Master Mix (Vazyme, Q811). The primers and probes for stem-loop qPCR are listed in Table 2

**TABLE 2:** Primers used for stem-loop qRT-PCR^*a*^

miRNA	Specific forward primer	RT specific stem-loop primer
miR-200a-3p	GCGCG**TAACACTGTCTGGTAA**	*GGTCGTATGCAAAGCAGGGTCCGAGGTATCCATCG CACGCATCGCACTGCATACGACC*ACATCG
miR-200b-3p	GCGCG**TAATACTGCCTGGTAA**	*GGTCGTATGCAAAGCAGGGTCCGAGGTATCCATCG CACGCATCGCACTGCATACGACC*TCATCA
miR-200c-3p	CGCG**TAATACTGCCGGGTAAT**	*GGTCGTATGCAAAGCAGGGTCCGAGGTATCCATCG CACGCATCGCACTGCATACGACC*TCCATC
miR-141-3p	GCGCG**TAACACTGTCTGGTAA**	*GGTCGTATGCAAAGCAGGGTCCGAGGTATCCATCG CACGCATCGCACTGCATACGACC*CCATCT
miR-429	CGCGCG**TAATACTGTCTGGTAA**	*GGTCGTATGCAAAGCAGGGTCCGAGGTATCCATCG CACGCATCGCACTGCATACGACC*ACGGTT

^*a*^ Boldface represents miRNA specific sequences. Italics represents stem-loop sequences. The miRNA complementary specific sequences are underlined. Universal TaqMan probe, FAM 5′TCCATCGCACGCATCGCACT- 3′ BHQ-1; universal reverse primer, GAGCAGGGTCCGAGGT; U6-F, CTCGCTTCGGCAGCACA; U6-R, AACGCTTCACGAATTTGCGT; U6-TaqMan probe, FAM 5′ TAGCATGGCCCCTGCGCAAG 3′ BHQ-1.

### Fluorescence *in situ* hybridization (FISH)

FISH probes labeled with the Quasar 570 fluorophore for detecting miR-200b-3p were purchased from GenePharma (Shanghai, China). 293T cells infected with IAV (MOI = 0.01, 24 h) or VSV (MOI = 0.01, 16 h) were fixed for 10 min with 4% paraformaldehyde for *in situ* hybridization analysis and then washed with PBS three times. Cells were permeabilized with 0.2% Triton X-100 for 10 min and washed briefly. Cells were then incubated with wash buffer A (SMF-WA1-60, Biosearch Technologies, Petaluma, CA, USA) for 5 min. Wash buffer A was removed, and the hybridization buffer (SMFHB1-10, Biosearch Technologies) containing FISH Probes (final concentration, 12.5 nM) were added and incubated for 16 h at 37°C in the dark. The hybridization buffer was then removed, and cells were incubated with wash buffer A for 5 min at 37°C. Cells were then stained with DAPI for 10 min at room temperature. Cells images were obtained with an EVOS FL Auto imaging system (Thermo Fisher Scientific) after being washed with PBS three times.

### Western blot

Cells were cultured in 24-well plates and lysed with NP40 lysis buffer (50 mM Tris-HCl (pH 7.5), 150 mM NaCl, 5 mM EDTA, and 0.5% NP-40) supplemented with a protease inhibitor cocktail and PMSF (1 mM) for 30 min at 4°C. The cell lysates were centrifuged for 10 min at 12,000 g and 4°C. The supernatants were transferred into a new tube, resolved by sodium dodecyl sulfate gel electrophoresis (SDS-PAGE), and transferred to PVDF membranes (Bio-Rad) to measure protein expression. The membrane was blocked with TBS-T buffer (10  mM Tris, pH 7.4, 150  mM NaCl, 0.1% Tween-20) containing 5% nonfat dry milk for 1  h at room temperature. Primary Antibody Dilution Buffer (Beyotime, P0023A-100ml) is used to dilute primary antibodies to working concentrations, then the membranes were incubated with primary antibodies at 4°C overnight. The HRP-conjugated goat antiMouse IgG secondary antibodies (Boster, Wuhan, China, BA1051) or HRP-conjugated goat antiRabbit IgG secondary antibodies (Boster, BA1055) were diluted in TBS-T (1:5,000 ratio). Next, the membranes were incubated with secondary antibodies for 1 h at room temperature. The blots were developed using the BeyoECL Plus (Beyotime, P0018S) and imaged with an Amersham Imager 600 (GE Healthcare) imaging system.

### Cellular fractionation

Cells were washed twice in ice-cold LS buffer (20 mM Hepes pH 7.8, 0.5 mM DTT, 0.5 mM MgCl_2_ in water) and allowed to swell on ice for 20 min. 293T cells were gently scraped and disrupted on ice. The lysates were centrifuged at 6,006 g for 2 min at 4°C to pellet the nuclei. The supernatant (cytoplasmic fraction) was removed. The nuclei were washed five times in PBS, placed in nuclei resuspension buffer (50 mM Tris-HCl pH 8, 0.5 mM MgCl_2_, 20 mM iodoactetamide supplemented with protease inhibitor (Roche) and sonicated. Proteins were resolved by SDS-PAGE and detected by Western blot.

### Construction of promoter-reporter plasmids

The miR-200b-3p promoter-reporter plasmids R5, R4, R3, R2, and R1 contain the corresponding nucleotides proximal promoter miR-200b-3p sequences were cloned by PCR amplification using genomic DNA of cells as templates and subsequently cloned into PGL3-basic (Promega, Madison, WI, USA). The miR-200b-3p promoter constructs containing site-specific mutation for the transcription factor binding site were constructed by overlap-expression PCR. Sequencing was used to verify all constructs. The miR-200b-3p promoter constructs were amplified using the following primers: R5 (chr 1: 1164586-1167085)- forward primer: 5′-GAGGGCTGCAAATCCACGCC-3′; R4 (chr1: 1165086-1167085) -forward primer：5′-TCCCGGCGACGGTTGACAAGAA-3′; R3 (chr1: 1165586-1167085) -forward primer：5′- CCGGGCCCTGAACCTGGC-3′; R2 (chr1: 1166086-1167085) -forward primer: 5′- CCACCTACAGCCTCTGTGGGT-3′; R1 (chr1: 1166585-1167085)-forward primer: 5′- CGGGGCAGCATGGGAGAG-3′; and Universal reverse primers：TGAGGG TTGCATGGGACTCGCT.

### Promoter activity assay

293T cells were co-transfected with 100 ng of full length, a series of truncated or mutant promoter firefly-luciferase reporter constructs, and 10 ng of Renilla luciferase vector (pRL-TK). Luciferase activities were determined with the Dual Luciferase Reporter Assay System (Promega) and expressed as relative luciferase activity by normalizing firefly luciferase activity against Renilla luciferase activity, according to the manufacturer’s protocol.

### Chromatin immunoprecipitation (ChIP)

ChromaFlash High-Sensitivity ChIP kit (Epigentek, Farmingdale, NY, USA) was used to perform ChIP assay according to the manufacturer’s protocol. Cells were infected with IAV (MOI = 0.01) for 36 h or not. The growth media of cells were removed, and cells were rinsed three times with cold PBS. Cells were added with formaldehyde to a final concentration of 1% and incubated at room temperature for 15 min. Glycine was added to cells to a final concentration of 125 mM to stop the cross-linking reaction and then sonicated to a fragment size range of 100–700 bp. Immunoprecipitation was performed by incubating sheared chromatin overnight at 4˚C with antiCREB antibody (CST, #9197) or rabbit IgG isotype (CST, #3900) and protein A + G Agarose beads (Santa Cruz Biotechnology, Cat# sc-2003). DNA precipitated from the samples was subjected to PCR amplification detecting a segment of miR-200b-3p promoter region using the primers: 5′- CCCAGGACCCAAAGCTGGTG-3′ (F) and 5′-AGTAAGATGGCCACGGCTGC-3′ (R). PCR products were resolved by 2% agarose gel electrophoresis and visualized using UV light. Input chromatin relative to its abundance was used to determine the expression level of a target DNA sequence.

### RNA immunoprecipitation (RIP)

RIP assay was performed as described previously (34). 293T cells were lysed in 0.5% NP-40, 150 mM KCl, and 25 mM tris-glycine (pH 7.5), and incubated with Flag affinity beads overnight. The lysate was then washed with 300 mM NaCl, 50 mM tris-glycine (pH 7.5), 5 mM MgCl_2_, and 0.05% NP-40. The TRIzol reagent was used according to the manufacturer’s protocol to extract RNA from immunoprecipitated RNA-proteins. Finally, collected RNA was reversed into cDNA and TBK1 mRNA was detected by qPCR by using primers: RIP-F: 5′- GCACAAGAAAATAACGCTTGGGCA-3′ and RIP-R: 5′- GGTCGGTCATGGCTTTCTTCTCG-3′.

### Northern blot

Northern blot hybridization was performed by a nonradioactive method. Locked nucleic acid (LNA)-modified probes were synthesized and 3′ end labeled with digoxigenin by Tsingke Biotech (Wuhan, China). RNAs of < 200 nucleotides were purified from cultured cells using MiPure Cell/Tissue miRNA Kit (Vazyme, RC201) following the manufacturer’s instructions. Samples of 20 mg of total RNA were analyzed using a 15% polyacrylamide gel and transferred to Hybond-N + nylon membranes. Hybridization of membranes with digoxigenin-labeled DNA probes was performed as previously reported (35). Signal detection was performed as described in the manual for a DIG High Prime DNA labeling and detection starter kit II (Roche, Switzerland). The probes used for Northern blot hybridizations are listed here, U6: CGaATtTGcGTgTCaTCcTTgC and miR-200b-3p: TcaTCaTTaCCaGGcAGtATtA, the lowercase letters represent LNA-modified nucleotide.

### Dual-luciferase reporter assays

Luciferase reporter vectors containing WT TBK1 3′ UTR or MUT TBK1 3′ UTR were co-transfected with the control mimic or miR-200b-3p mimic into HEK-293T cells to validate the miR-200b-3p targeting TBK1. The transfected cell lysates were analyzed by using the dual luciferase assay kit (Promega) at 24 h post-transfection. All obtained luciferase values were normalized against the Renilla luciferase control.

HEK-293T cells grown in 48-well plates were co-transfected with luciferase reporter plasmids (IFN β-Luc, IRF3-Luc, ISRE-Luc, or NF-κB-Luc) and the pRL-TK plasmid to detect activation of the IFN pathway, along with the indicated amount of empty vector or miRNAs. 293T cells were left untreated or were treated with SeV for additional 12 h. The dual luciferase assay kit (Promega) was used to prepare and analyze cell lysates for firefly and Renilla luciferase activities.

### Confocal microscopy

HEK-293T cells seeded on 14 mm coverslips were transfected with miRNAs or infected with SeV or IAV. 293T cells were fixed with 4% paraformaldehyde, permeabilized with 0.1% Triton X-100, and then stained with antibodies against CREB, IAV-NP, VSV-G, SeV-HN, IRF3, p65, or DAPI after incubation. 293T cells were incubated with Alexa 488-conjugated or 594-conjugated secondary antibodies for 1 h at room temperature after being washed three times. Staining was visualized with a ZEISS LSM 880 confocal microscope under an oil objective (Carl Zeiss AG, Oberkochen, Germany).

### Mouse infection

The 6-week-old female C57BL/6 mice were randomly divided into indicated groups and infected intranasally (i.n.) with 200 plaque-forming units (PFU) of IAV or 1 × 10^7^ FFU VSV or mock infected with DMEM in a volume of 20 µL. Changes in the mice’s body weight and mortality were monitored daily. Mice that lost more than 25% of initial weight were humanely euthanized with CO_2_. The brains and lungs were collected for qPCR, histopathology, or immunohistochemistry analysis.

### ELISA

ELISA was performed to quantify the amount of IFN-β in the 293T cell culture supernatants. Commercially available Human IFN-β ELISA kits (RayBiotech, Atlanta, GA, USA) were used following the manufacturer’s instructions.

### Histopathological analysis and immunohistochemistry (IHC)

PBS was used to perfuse the mice intracardially. Brains or lungs were removed and placed in 4% paraformaldehyde at room temperature for 12 h. After dehydration and wax immersion, samples were embedded in paraffin and sectioned into 4 µm. Sections were stained with hematoxylin and eosin (H&E) for histopathological analysis. Sections were processed using antigen retrieval and endogenous peroxidase quenching followed by anti-IAV-NP or anti-VSV-G antibody staining for the immunohistochemical analysis.

### Statistical analysis

Data were expressed as the mean and standard deviation (SD). A student’s *t*-test was performed to analyze the significant differences between the two groups. The log-rank (Mantel-Cox) test was used to analyze the survival ratio. The asterisks indicate statistical significance (*, *P* < 0.05; **, *P* < 0.01; ***, *P* < 0.001). GraphPad Prism software, version 8.0 (GraphPad Software, La Jolla, CA, USA) was used to analyze and plot graphs.

## RESULTS

### MiR-200b-3p is upregulated in IAV or VSV-infected cells

**Fig 1:**
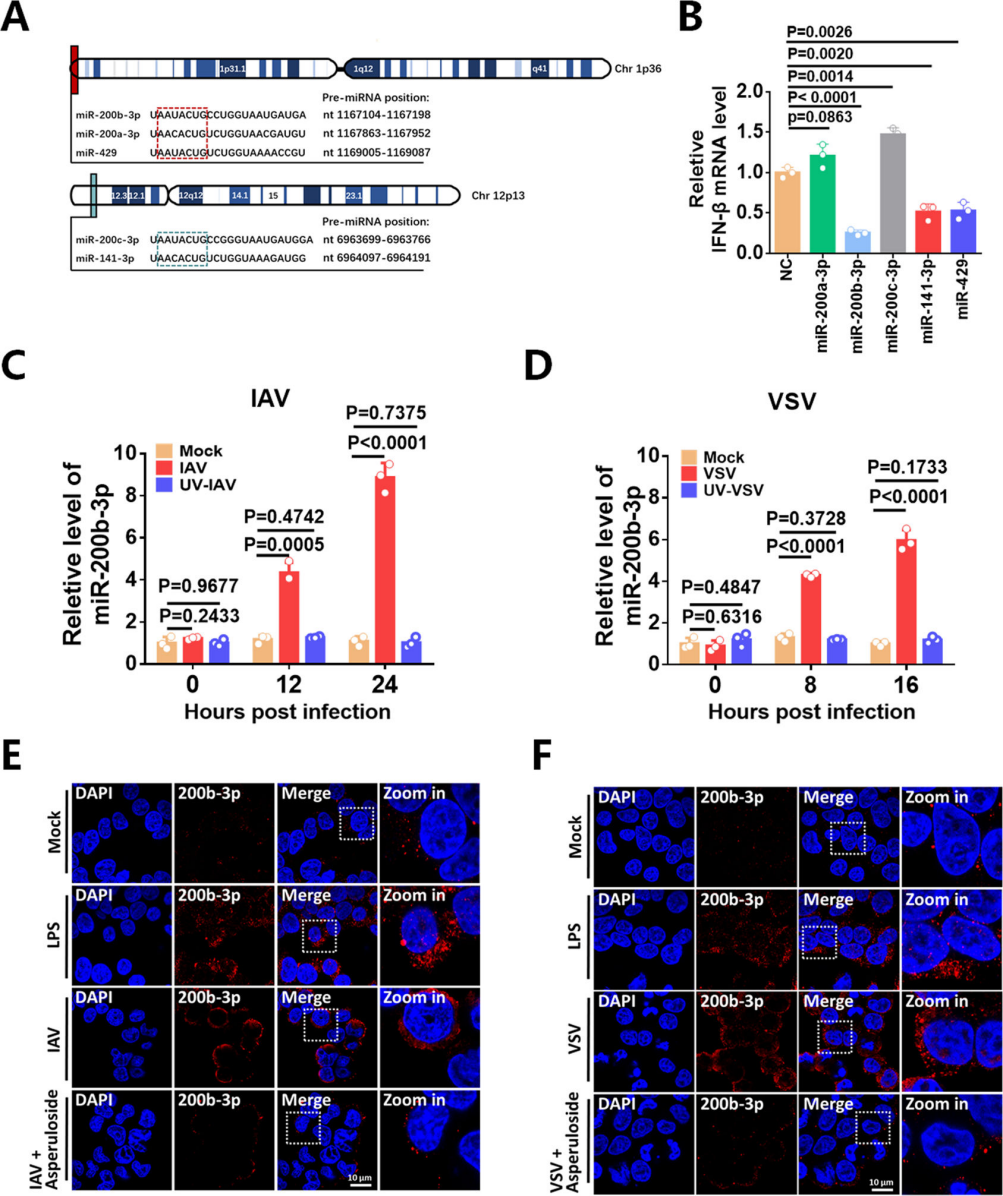
MiR-200b-3p expression is upregulated in virus-infected cells. (**A**) Schematic representation of the genomic loci of the miR-200 family in humans. Chromosomal loci, locations of pre-miRNA hairpins, and sequences of mature miRNAs are indicated. Information was obtained from Sanger miRbase (38). (**B**) 293T cells were individually transfected with NC mimics, miR-200a-3p mimics, miR-200b-3p mimics, miR-200c-3p mimics, miR-141-3p mimics, or miR-429 mimics, and then infected with SeV for 12 h before qPCR analysis. (**C**) 293T cells were infected with IAV at an MOI of 0.01 or incubated with UV-irradiated inactive IAV for the indicated time periods. Stem-loop qPCR was performed to detect the expression of miR-200b-3p. (**D**)293T cells were infected with VSV at an MOI of 0.01 or incubated with UV-irradiated inactive VSV. Then 293T cells were collected at 8 and 16 h postinfection and analyzed by stem-loop qPCR analysis. (**E**) Detection of miR-200b-3p in 293T cells infected with IAV at MOI of 0.01 for 24 h or treated with LPS (100 ng, 9 h) or infected with IAV at MOI of 0.01 and then treated with asperuloside (20 mM, 12 h) using FISH. (**F**) Detection of miR-200b-3p in 293T cells infected with VSV at MOI of 0.01 for 16 h or treated with LPS (100 ng, 9 h) or infected with VSV and then treated with asperuloside (20 mM, 12 h) by FISH. miR-200b-3p (red) and nucleus (blue). Student’s t-test was used for statistical analysis of comparisons between groups. Bar graph shows the means ± SD, n = 3. Scale bar = 10 µm.

The miR-200 family consists of five evolutionarily conserved members: miR-200a-3p, miR-200b-3p, miR-200c-3p, miR-141-3p, and miR-429. MiR-200a-3p, miR-200b-3p, and miR-429 are located on chromosome 1, whereas miR-200c-3p and miR-141-3p are located on chromosome 12. In addition, the seed sequences (nt 2-8) of the human miR-200 miRNAs are identical except for an alternative U or C at the third position. This sequence variation divides the family members into two groups: miR-200b, miR-200c, and miR-429, with U in the seed sequence, and miR-200a and miR-141, with C in the seed sequence (Fig. 1A) (36). A recent report showed that miR-200c reduced IAV replication by promoting IFN-I production (37). Considering the highly conserved seed sequences, we hypothesized that the five members of the miR-200 family may play a role in regulating IFN-I production during viral infection. Therefore, we separately expressed the five members (miR-200a-3p, miR-200b-3p, miR-200c-3p, miR-141-3p, and miR-429) in Sendai virus (SeV)-infected 293T cells, which robustly stimulated IFN-I production. Interestingly, transfection with miR-200a-3p and miR-200c-3p mimics upregulated IFN-β transcription, whereas transfection with miR-200b-3p, miR-141-3p, and miR-429 mimics strongly inhibited IFN-β mRNA transcription (Fig. 1B). Notably, the upregulation of miR-200c-3p on IFN-β production is consistent with the previous report (37).

Since miR-200b-3p displayed the most pronounced inhibitory effect on IFN-β expression among the miR-200 family, we then focused on miR-200b-3p in the following study. We used stem-loop qRT-PCR (stem-loop qPCR) assay to detect miR-200b-3p, which is highly accurate for miRNA detection, and only 0.1–3.7% nonspecific signal was observed for the miRNAs that differed by a single nucleotide (33, 39, 40). To further prove that our detection of miR-200b-3p is specific and does not detect any other miR-200 family members, we separately transfected cells with miR-200a/200b/200 c/141/429 mimics and then used primers targeting miR-200b-3p for stem-loop qPCR analysis. The results showed that the primers targeting miR-200b-3p could specifically detect miR-200b-3p but not other miR-200 family members (Fig. S1A).

We then examined miR-200b-3p levels in 293T cells infected with IAV or VSV at different time points by stem-loop qPCR to characterize miR-200b-3p expression upon viral infection. The results showed that mature miR-200b-3p was constitutively expressed in cells and increased significantly in a time-dependent manner after IAV or VSV infection (Fig. 1C and 1D). Cells inoculated with UV-inactivated IAV or VSV showed no significant change in the expression level of miR-200b-3p (Fig. 1C and D), suggesting that miR-200b-3p expression was triggered by viral replication. In addition, miR-200b-3p increased in a dose-dependent manner upon IAV or VSV infection at different MOIs (Fig. S1B and C). We also used the Northern blot assay and showed that IAV or VSV infection increased the expression of mature miR-200b-3p in a time-dependent manner (Fig. S1D and G). We then measured the expression of the primary miR-200b-3p transcript (pri-miR-200b-3p) and the miR-200b-3p precursor (pre-miR-200b-3p) in virus-infected cells, from which the mature miR-200b-3p is processed. As expected, the expression of pri-miR-200b-3p and pre-miR-200b-3p showed a time-dependent increase in cells after IAV (Fig. S1E and F) or VSV (Fig. S1H and I) infection. These results showed that the induction of pri-miR-200b-3p and pre-miR-200b-3p was coincided with the induction of mature miR-200b-3p during virus infection, suggesting that the upregulation of miR-200b-3p occurred at the transcriptional level.

We then examined the effects of the classical inflammatory activator LPS on miR-200b-3p, as transcriptional regulatory effects of inflammatory pathways on microRNAs have been widely reported (16, 41). To demonstrate the specificity of the probe for miR-200b-3p, we separately transfected miR-200 family members’ mimics into cells and then detected by Fluorescence *in situ* hybridization (FISH). The results in Fig. S2A showed that the probes targeting miR-200b-3p could only specifically detect miR-200b-3p. FISH assay was further used and demonstrated that miR-200b expression was increased in IAV-infected or LPS-treated cells compared to mock-treated cells (Fig. 1E). Similarly, stimulation by LPS or VSV infection caused a significant increase of miR-200b-3p in 293T cells (Fig. 1F). Meanwhile, the application of asperuloside (42), a broad-spectrum inflammatory inhibitor, significantly downregulated IAV- or VSV-induced expression of in miR-200b-3p in cells (Fig. 1E and F). Meantime, we used confocal microscopy to confirm IAV and VSV infection under the same experimental conditions (Fig. S2B and C). Taken together, the above results suggested that IAV and VSV infection upregulated the miR-200b-3p expression through activation of inflammatory pathways.

### IAV infection upregulates miR-200b-3p through ERK and p38 pathways

**Fig 2:**
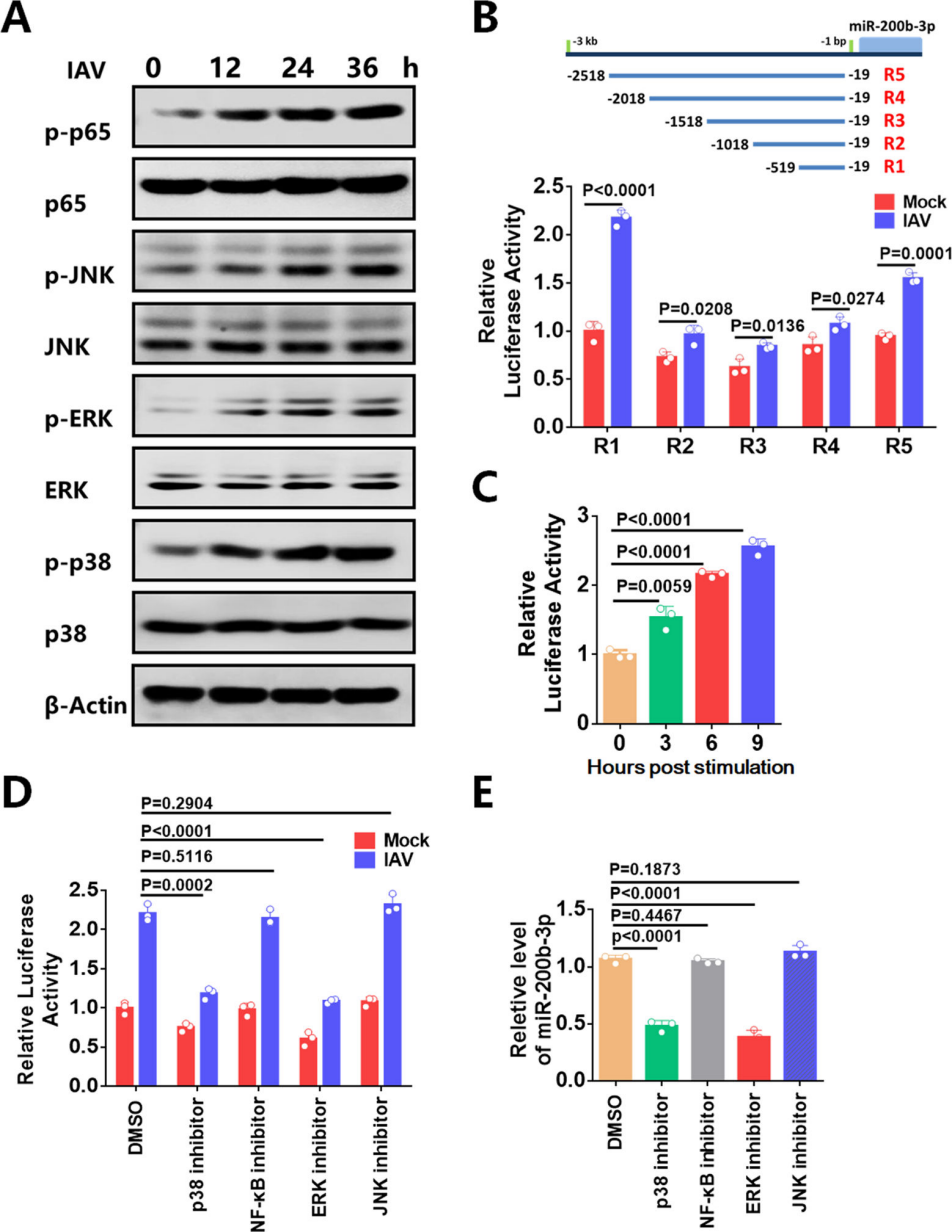
Analysis of signaling pathways involved in the regulation of miR-200b-3p expression upon IAV infection. (**A**) 293T cells were infected with IAV at an MOI of 0.01 and harvested at the indicated time points. Western blotting was used to confirm the expression and phosphorylation of the p65, JNK, ERK, and p38 in 293T cells. (**B**) The promoter-reporter constructs (100 ng) and PRL-TK (10 ng) were co-transfected into 293T cells. At 24 h after transfection, cells were mock infected with DMEM or infected with IAV at an MOI of 0.01 for another 24 h. Samples were collected and analyzed for dual luciferase activity. Results were plotted as firefly luciferase activity/Renilla luciferase activity and expressed as normalization to R1 incubated with DMEM. (**C**) Luciferase promoter-reporter plasmids R1 and the pRL-TK plasmid were co-transfected into 293T cells. 293T cells were treated with LPS for the indicated times before reporter assays at 24 h after transfection. (**D**) 293T cells were transfected with R1 promoter for 24 h and then either left uninfected or infected with IAV at an MOI of 0.01, and then treated with signaling pathway-specific inhibitors (p38 inhibitor, 1 mM, 24 h; NF-kB inhibitor, 5 mM, 24 h; JNK inhibitor, 0.5 mM, 12 h; or ERK inhibitor, 5 mM, 24 h) or dinethyl sulfoxide (DMSO). Results were plotted as firefly luciferase activity/Renilla luciferase activity and expressed as normalization to R1 incubated with DMSO. (**E**) 293T cells were infected with IAV at an MOI of 0.01 and then respectively treated with signaling pathway-specific inhibitors (p38 inhibitor, 1 mM, 24 h; NF-kB inhibitor, 5 mM, 24 h; JNK inhibitor, 0.5 mM, 12 h; or ERK inhibitor, 5 mM, 24 h) or DMSO, respectively, and the levels of miR-200b-3p expression were detected by stem-loop qPCR. All results are represented as fold change of the group treated with DMSO, which was considered as 1. Student’s t-test was used for statistical analysis of comparisons between groups. Bar graph shows the means ± SD, n = 3. Western blot data were representative of at least three independent experiments.

We then sought to determine the exact inflammatory pathway(s) responsible for the regulation of miR-200b-3p expression after confirming that inflammatory pathway activation was involved in the upregulation of miR-200b-3p. We found that IAV infection promoted the phosphorylation levels of p65, c-Jun N-terminal kinase (JNK), extracellular signal-regulated kinases 1 and 2 (ERK), and p38 in a time-dependent manner (Fig. 2A), suggesting that IAV infection activated NF-κB and mitogen-activated protein kinase (MAPK) pathways, which is consistent with a previous report (43). The signal transduction pathways mediated by NF-κB and MAPK contributed to the activation of transcription factors (16, 41). Transcription factors bind to miRNA regulatory elements and further regulate miRNA expression.

To investigate the transcriptional regulation of miR-200b-3p, we analyzed the promoter region of miR-200b-3p. An approximately 2.5 kb DNA fragment containing noncoding sequences upstream of miR-200b-3p stem loop as the putative promoter. Luciferase reporter genes with different lengths of the miR-200b-3p putative promoter region were constructed and transfected into cells to determine basal and IAV-induced promoter activity. The region 5 (R5), (chr1: 1164586-1167085) showed basal promoter activity and clear IAV inducibility, as shown in Fig. 2B, which was reduced by three truncations of this full-length region: region 4 (R4), (chr1: 1165086-1167085); region 3 (R3), (chr1: 1165586-1167085); and region 2 (*R2*), (chr1: 1166086-1167085). However, the reporter gene region 1 (R1), (chr1: 1166585-1167085) had a higher luciferase activity in both mock-infected and IAV-infected cells, comparable to that of the longest reporter R5, suggesting that R1 had fully intact promoter activity.

We then treated cells with LPS for the indicated time periods, and the R1 reporter activity of miR-200b-3p was confirmed to be upregulated (Fig. 2C), suggesting that the R1 reporter activity of miR-200b-3p was regulated by the activation of inflammatory signaling pathways. Specific inhibitors of these signaling pathways were then used to analyze which inflammatory signaling pathway plays an important role in the IAV-induced expression of miR-200b-3p. The results of the luciferase activity assay showed that IAV-induced miR-200b-3p promoter activity was significantly reduced in cells treated with p38-specific or ERK-specific inhibitor compared to DMSO-treated cells (Fig. 2D). In contrast, cells treated with NF-κB-specific or JNK-specific inhibitors had no apparent inhibitory effect (Fig. 2D). Stem-loop qPCR analysis also showed that either the p38-specific inhibitor or the ERK-specific inhibitor significantly downregulated IAV-induced miR-200b-3p (Fig. 2E). Furthermore, stem-loop qPCR results showed that overexpression of p38 or ERK upregulated the expression of miR-200b-3p (Fig. S3). Taken together, these results indicate that IAV infection upregulates miR-200b-3p through the ERK and p38 signaling pathways.

### CREB regulates miR-200b-3p promoter activity

**Fig 3:**
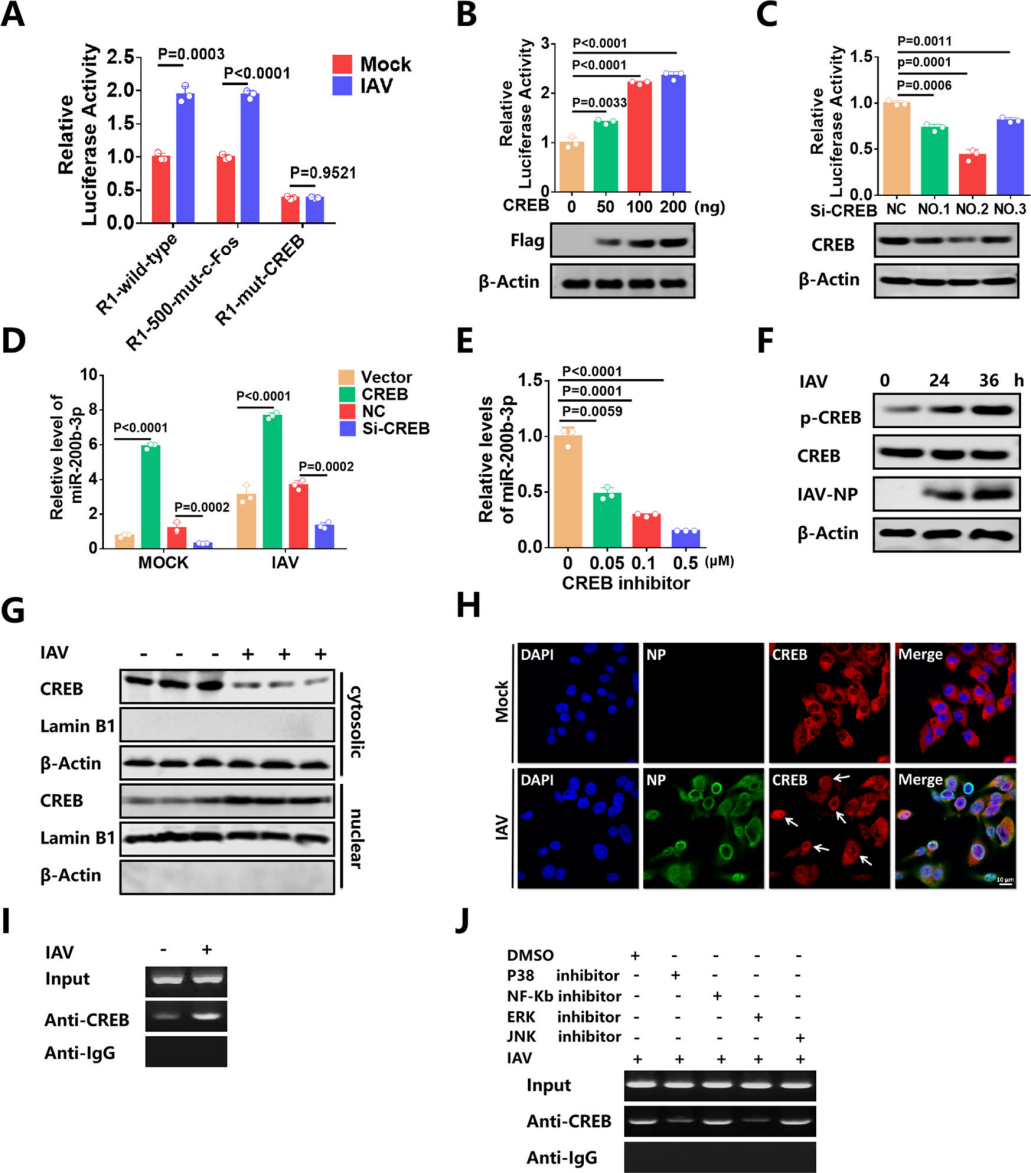
CREB elements regulate miR-200b-3p promoter activity. (**A**) The R1 and R1-mut reporter constructs were co-transfected into 293T cells with PRL-TK, respectively. At 24 h after transfection, cells were infected with IAV at an MOI of 0.01 or mock infected for 24 h. Samples were collected and analyzed for dual luciferase activity. (**B**) 293T cells were co-transfected with R1 reporter plasmid, PRL-TK vector, and different concentrations of Flag-tagged CREB expressing plasmid for 24 h. Dual luciferase assays measured the miR-200b-3p promoter activities. (**C**) Luciferase reporter plasmids (**R1**) and the pRL-TK plasmid were co-transfected into 293T cells together with CREB siRNA or NC. Western blots showed the expression levels of CREB at the bottom. (**D**) CREB expression plasmid or siRNA was transfected into 293T cells. At 24 h after transfection, cells were left uninfected or were infected with IAV at an MOI of 0.01 for 24 h before stem-loop qPCR assays. (**E**) 293T cells were infected with IAV at an MOI of 0.01, and then treated with different concentrations of CREB-specific inhibitors for 24 h, the miR-200b-3p expression levels were detected by stem-loop qPCR. (**F**) 293T cells were infected with IAV at an MOI of 0.01 for the indicated time periods. The expression of phosphorylated CREB, total CREB, and β-Actin in the whole cell lysates was monitored by the Western blot. (**G**) The cytosolic extracts (upper panel) and nuclear extracts (lower panel) were isolated and subjected to Western blot with Abs against CREB, Lamin B1, and β-Actin in 293T cells. Lamin B1 was used as a marker for nuclei. β-Actin and Lamin B1 were used as loading controls. (**H**) 293T cells were mock-infected (top) or infected with IAV virus (bottom) at an MOI of 0.01 for 36 h. 293T cells were fixed, and cell nuclei (left), NP (middle), and CREB (right) were stained as described in the text. (**I**) 293T cells were infected with IAV at an MOI of 0.01 for 36 h prior to ChIP assays. Fixed chromatin from 293T cells was prepared and immunoprecipitated with antiCREB antibody or normal IgG. ChIP primers were designed to amplify the region containing CREB binding sites in the miR-200b-3p promoter. PCR products were separated by acrylamide gel electrophoresis. (**J**) AntiCREB antibody or control IgG was used for chromatin immunoprecipitation. The upper panel shows the input DNA amplification before immunoprecipitation. Student’s t-test was used for statistical analysis of comparisons between groups. Bar graph shows the means ± SD, n = 3. Scale bar = 10 µm. Western blot data are representative of at least three independent experiments.

To further clarify which transcription factor controls the expression of miR-200b-3p, CONSITE and JASPAR software were used for bioinformatics analyses to predict potential transcription factor-binding sites in the miR-200b-3p promoter (R1) (44, 45). The putative binding sequences for two potential transcription factors, CREB and c-Fos, which are involved in the MAPK pathway (26), were found within the miR-200b-3p promoter region (R1). To determine whether the predicted transcription factor was responsive to the regulation of miR-200b-3p, a series of promoter constructs, including wild type (R1-wild-type), CREB binding site mutation (R1-mut-CREB) (TGTCCTCA to ACTGCAGT), and c-Fos binding site mutation (R1-mut-c-Fos) (CTGCCTCA to CACCAAGT), were generated and tested for their promoter activity under IAV infection. The results showed that mutation of the c-Fos binding site had no significant effect on constitutive or inducible luciferase activity (Fig. 3A). However, R1 with mutation of the CREB binding site showed significantly less response to IAV infection compared with the wild type, suggesting that the activation of miR-200b-3p promoter by IAV infection requires CREB (Fig. 3A). Furthermore, the overexpression of CREB significantly enhanced the promoter activity and the amount of miR-200b-3p (Fig. 3B and 3D), whereas knockdown of CREB by using siRNAs had a suppressive effect (Fig. 3C and 3D). Also, the CREB-specific inhibitor could inhibit the expression of miR-200b-3p in a dose-dependent manner (Fig. 3E).

Since phosphorylated transcription factors are transported to the nucleus and bind to the corresponding DNA fragments to exert their functions, we then tested whether IAV infection promoted the phosphorylation and nuclear translocation of CREB. Western blot results showed that IAV infection promoted CREB phosphorylation in a time-dependent manner (Fig. 3F). Consistently, Western blot results showed that the translocation of CREB from the cytoplasm to the nucleus increased upon IAV infection (Fig. 3G). In addition, confocal microscopy analysis showed that CREB translocated into the nucleus after IAV infection (Fig. 3H). Chromatin immunoprecipitation assay (ChIP) was then performed and confirmed that IAV infection could increase the binding of CREB to the miR-200b-3p promoter (Fig. 3I). Furthermore, the binding of CREB to the miR-200b-3p promoter was attenuated by ERK inhibitor and p38 inhibitor upon IAV infection, while the binding activity remained unchanged in cells treated with NF-κB inhibitor or JNK inhibitor (Fig. 3J). This result is consistent with CREB acting as a transcription factor downstream of the ERK and p38 pathways. In addition, Western blot results showed that UV-inactivated IAV and VSV (UV-IAV and UV-VSV) did not activate p38, ERK pathway, and downstream CREB (Fig. S4A and B), which is consistent with the data that UV-IAV and UV-VSV did not upregulate miR-200b-3p expression. These results demonstrate that CREB plays an important role in IAV- or VSV-induced transcriptional regulation of miR-200b-3p.

### MiR-200b-3p directly targets 3′ UTR of TBK1 mRNA

**Fig 4:**
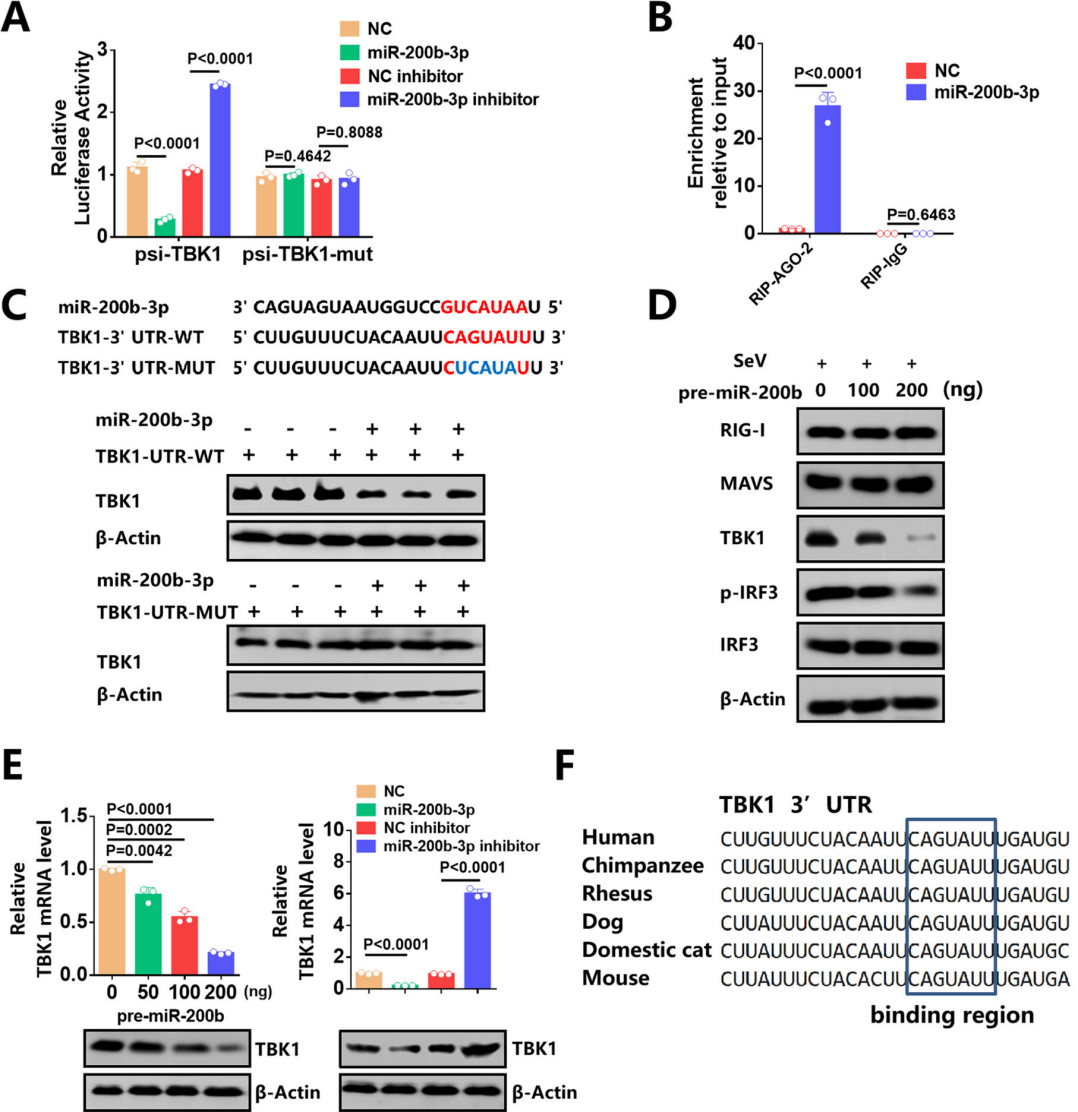
MiR-200b-3p directly targets TBK1 3′ UTR mRNA. (**A**) 293T cells were transfected with miR-200b-3p mimics, miR-200b-3p inhibitors, their control oligonucleotides, and a WT or mutated TBK1 3′ UTR luciferase reporter plasmid and measured for luciferase activity at 36 h after transfection. Data are presented as relative Renilla luciferase activities normalized to the value of firefly luciferase. Similar results were obtained in three independent experiments. (**B**) 293T cells were transfected with Flag-Ago2 in the presence of either the miR-200b-3p mimic or the NC mimic. 293T cells were subjected to RIP assay with an anti-Flag antibody after 36 h. TBK1 mRNA levels were quantified by using a qPCR assay. (**C**) Predicted miR-200b-3p binding sites in the 3′ UTR of the TBK1 mRNA. Perfect matches in seed regions are indicated in red. Mutations (indicated in blue) were generated in the binding sites of the 3′ UTR of the TBK1 mRNA. 293T cells were co-transfected with miR-200b-3p mimics and TBK1 expression vector with WT or mutant 3′ UTR for 36 h and were subjected to Western blot. (**D**) 293T cells were transfected with different concentrations of pre-miR-200b-3p expressing plasmid for 24 h and then stimulated with SeV for 12 h. The levels of RIG-I, MAVS, p-IRF3, IRF3, TBK1, and β-Actin were detected by Western blot. (**E**) 293T cells were transfected with pre-miR-200b-3p expressing plasmid or miR-200b-3p mimics or miR-200b-3p inhibitors for 36 h, TBK1 protein levels and mRNA levels were measured by Western blot and qPCR, respectively. (**F**) Alignment of TBK1 3′ UTR from different species for the miR-200b-3p binding region. Student’s t-test was used for statistical analysis of comparisons between groups. Bar graph shows the means ± SD, n = 3. Western blot data are representative of at least three independent experiments. SeV was used at a final concentration of 100 hemagglutinin units per ml.

To investigate the potential mechanism of miR-200b-3p in the regulation of IFN-I production, we examined its specific target. Using TargetScan 7.0 and miRanda, miRNA target-prediction algorithms (46 - 48), the 3′ UTR of TBK1 mRNA showed a potential putative target site for miR-200b-3p. Since TBK1 is essential for the production of IFN-I, we focus on the targeting of miR-200b-3p to TBK1. We co-transfected cells with miR-200b-3p mimic and wild type TBK1 3′ UTR luciferase reporter plasmid and found that luciferase levels were significantly reduced. In contrast, application of miR-200b-3p inhibitor resulted in a significant increase in luciferase activity (Fig. 4A). Furthermore, a 4 bp mutation in the miR-200b-3p target sequence abolished the negative effect of miR-200b-3p on the expression of the TBK1 3′ UTR reporter construct (Fig. 4A). Also, to prove the targeting specificity of miR-200b-3p mimic and inhibitor, we transfected miR-200b-3p mimic and inhibitor into cells separately, and then measured the levels of miR-200b family members. The stem-loop qPCR results showed that cells transfected with miR-200b-3p mimic only significantly upregulated the amount of miR-200b-3p, while cells transfected with miR-200b-3p inhibitor only significantly downregulated the amount of miR-200b-3p (Fig. S5A through E ).

We then performed an RNA immunoprecipitation (RIP) assay using Flag-tagged Ago2, which is a commercial component of the miRNAs-silencing complex (34), to confirm that TBK1 mRNA directly interacts with miR-200b-3p. The results showed that the TBK1 mRNA was significantly enriched in the miR-200b-3p group (Fig. 4B). Cells were co-transfected with miR-200b-3p mimics and TBK1 expression vector with WT or mutated 3′ UTR, and the expression of TBK1 in 293T cells was then measured. As expected, overexpression of miR-200b-3p reduced TBK1 levels in cells transfected with WT 3′ UTR TBK1, whereas TBK1 with mutated 3′ UTR was unaffected by miR-200b-3p (Fig. 4C). These results suggest that the nucleotide sequence in the TBK1 3′ UTR is a targeting site by miR-200b-3p.

We then transfected cells with pre-miR-200b-3p plasmid and infected them with SeV to confirm whether miR-200b-3p regulates endogenous TBK1. Western blot results showed that the pre-miR-200b-3p plasmid had a dose-dependent inhibitory effect on the protein level of TBK1 and the phosphorylation of IRF3, the downstream innate immune effector of TBK1, while the pre-miR-200b-3p plasmid had no effect on the expression of RIG-I and MAVS, which are the upstream effectors of TBK1 (Fig. 4D). Also, the pre-miR-200b-3p plasmid had a significant inhibitory effect both on the mRNA and protein levels of TBK1. Similarly, the applications of miR-200b-3p mimic suppressed TBK1 protein and mRNA levels, and the inhibition of TBK1 was restored in the presence of miR-200b-3p inhibitors (Fig. 4E). Interestingly, like most known and functionally well-defined miRNA binding sites, the miR-200b-3p binding site within the TBK1 3′ UTR is highly conserved in vertebrates (Fig. 4F). Taken together, these data suggest that TBK1 mRNA is a direct target of miR-200b-3p and TBK1 expression is regulated by miR-200b-3p.

### MiR-200b-3p negatively regulates IRF3 and NF-κB signaling pathway

**Fig 5:**
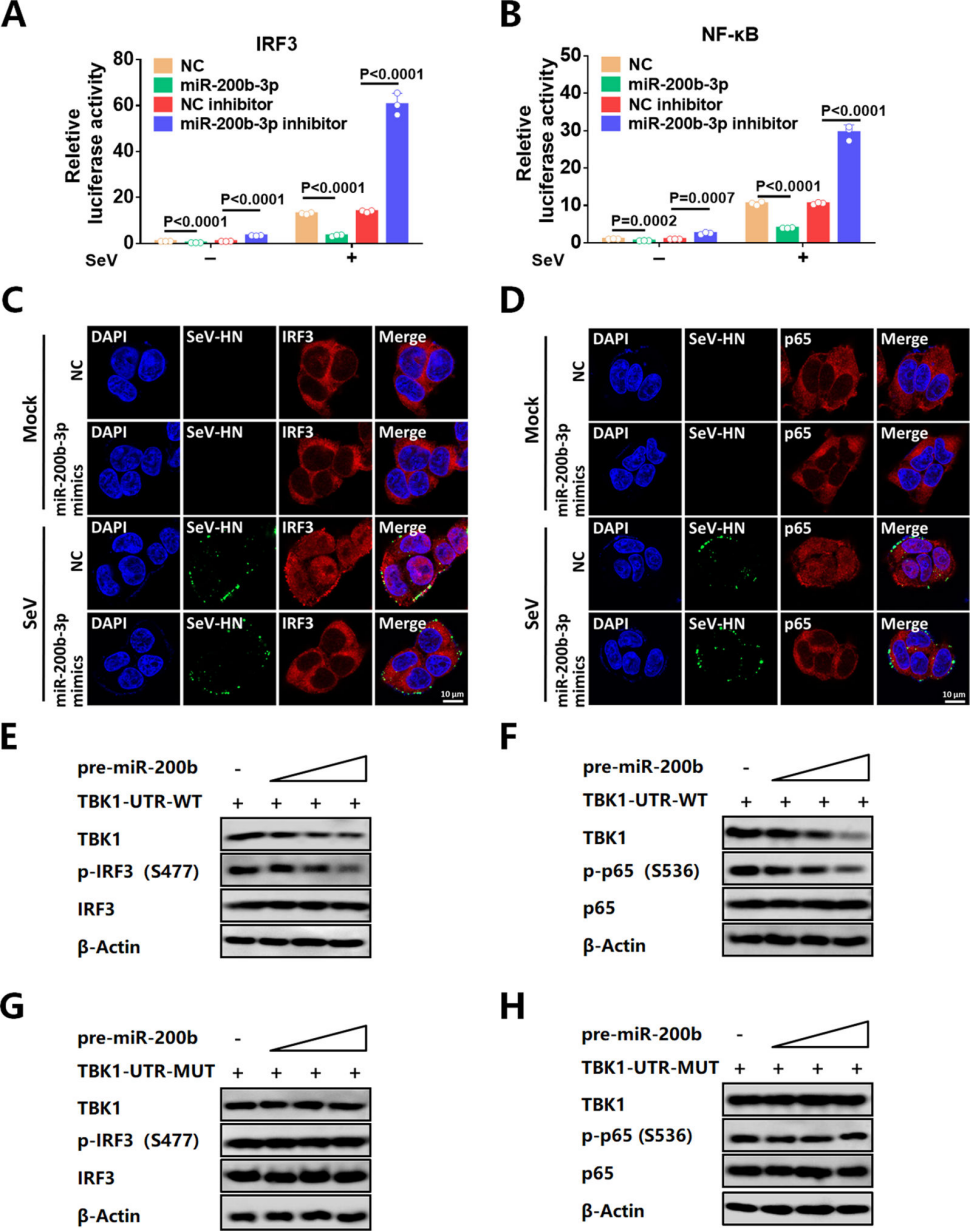
MiR-200b-3p downregulates the IRF3 and NF-κB signaling pathways through inhibition of TBK1. (**A, B**) Luciferase reporter plasmids (IRF3-Luc, NF-κB-luc) and pRL-TK plasmids were co-transfected into 293T cells, together with miR-200b-3p mimics or miR-200b-3p inhibitors. At 24 h after transfection, cells were left uninfected or infected with SeV for 12 h prior to reporter assays. (**C, D**) 293T cells were transfected with miR-200b-3p mimics or NC mimics and then left uninfected or infected with SeV for 12 h for confocal analysis. (**C**) IRF3 (red), SeV-HN (green), and nucleus (blue). (**D**) p65 (red), SeV-HN (green), and nucleus (blue). (**E, G**) 293T cells were co-transfected with different doses of pre-miR-200b-3p expression vector and WT 3′ UTR TBK1 (**E**) or mutant 3′ UTR TBK1 (**G**) for 36 h. Levels of TBK1, p-IRF3, IRF3, and β-Actin proteins in the whole-cell lysates were detected by Western blot. (**F, H**) 293T cells were co-transfected with different doses of pre-miR-200b-3p expression vector and WT 3′ UTR TBK1 (**F**) or mutant 3′ UTR TBK1 (**H**) for 36 h. The levels of TBK1, p-p65, p65, and β-Actin proteins in the whole-cell lysates were detected by Western blot. Student’s t-test was used for statistical analysis of comparisons between groups. Bar graph shows the means ± SD, n = 3. Scale bar = 10 µm. Western blot data are representative of at least three independent experiments. SeV was used at a final concentration of 100 hemagglutinin units per mL.

During viral infection, IRF3 and NF-κB can be phosphorylated by the protein kinase TBK1 and subsequently translocated to the nucleus as activated transcription factors (9, 49). Therefore, we investigated whether miR-200b-3p affects the IRF3 and NF-κB signaling pathways by targeting TBK1. The dual-luciferase reporter assays showed that miR-200b-3p mimics significantly impaired the activity of IRF3 and NF-κB, while the administration of miR-200b-3p inhibitor promoted the activity (Fig. 5A and B). We also tested and confirmed that transfection of pre-miR-200b-3p plasmid could downregulate the activation of IRF3 and NF-κB in a dose-dependent manner (Fig. S6A and B). The effect of miR-200b-3p on the nuclear translocation of endogenous IRF3 and NF-κB was then assessed by Western blot. MiR-200b-3p mimics transfection attenuated SeV-induced nuclear translocation of IRF3 and NF-κB p65 subunits (Fig. S6C and D). Consistently, we used confocal microscopy analysis and found reduced nuclear translocation of IRF3 and NF-κB p65 subunit in miR-200b-3p-overexpressed cells upon SeV infection (Fig. 5C and D). Furthermore, Western blot results showed that miR-200b-3p also suppressed the phosphorylation of IRF3, IRF7, p65, and ikbα (Fig. S6E and F). Correspondingly, treatment with miR-200b-3p inhibitor significantly increased the phosphorylation of IRF3, IRF7, p65, and ikbα in cells (Fig. S6E and F).

To further confirm that the regulation of IRF3 and p65 by miR-200b-3p was associated with TBK1, cells were co-transfected with miR-200b-3p mimics and TBK1 expression vectors with WT or mutated 3′ UTR, and the phosphorylation of IRF3 and p65 were determined. As shown in Fig. 5E and F, transfection with pre-miR-200b-3p plasmid had a dose-dependent inhibitory on the phosphorylation of IRF3 and p65. Consistently, the mutant 3′ UTR of TBK1 rescued the inhibition of phosphorylation (Fig. 5G and H). Taken together, these results suggest that miR-200b-3p inhibits the activation of IRF3 and NF-κB signaling pathways by targeting and degrading TBK1 mRNA.

### MiR-200b-3p inhibits TBK1-mediated type I interferon production

**Fig 6:**
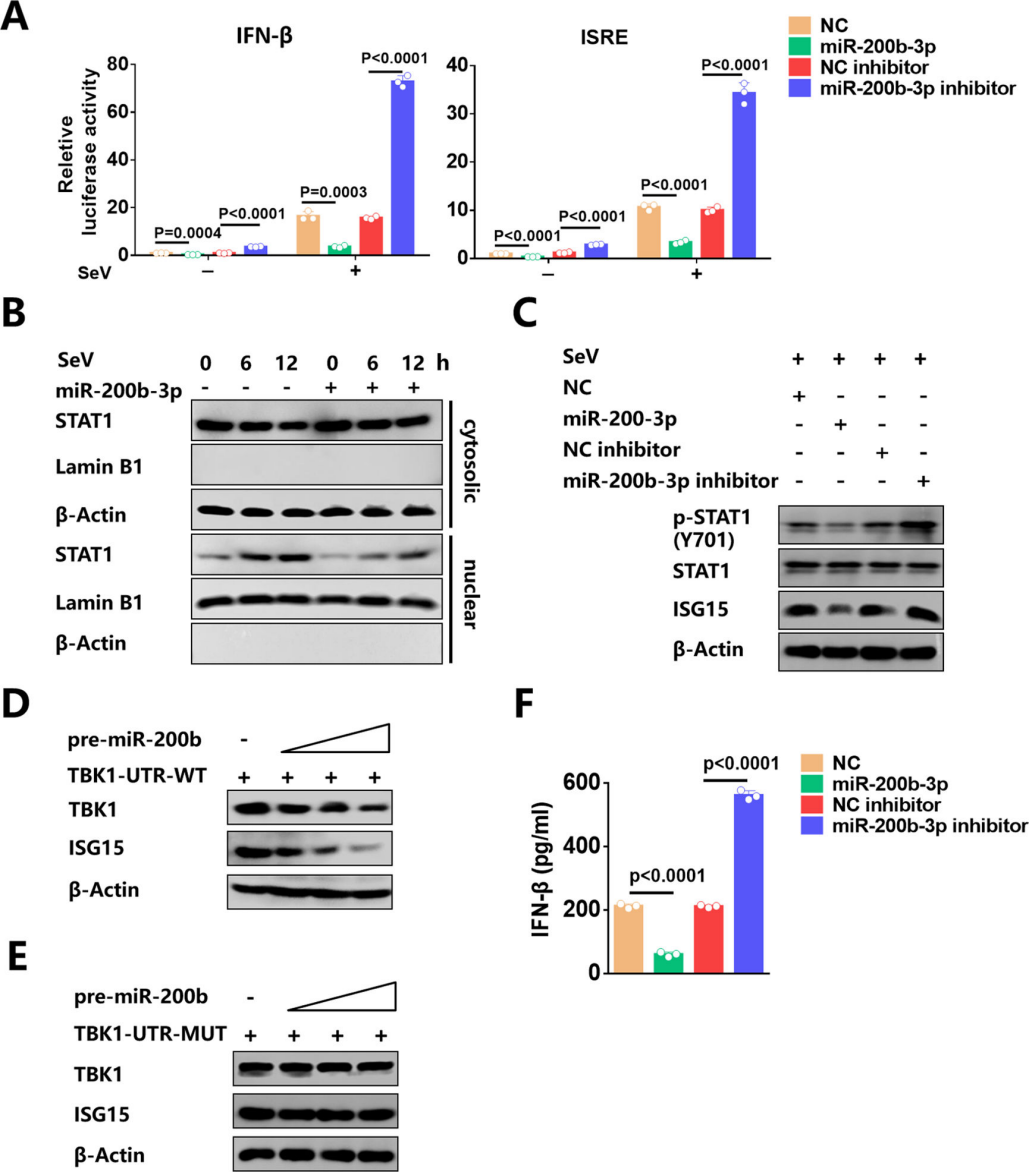
Inhibition of miR-200b-3p enhances IFN-I production. (**A**) Luciferase reporter plasmids (IFN or ISRE-Luc) and the pRL-TK plasmid were co-transfected into 293T cells, together with miR-200b-3p mimics or miR-200b-3p inhibitors. At 24 h after transfection, cells were left uninfected or were infected with SeV for 12 h prior to reporter assays. (**B**) 293T cells were transfected with miR-200b-3p mimics or NC mimics for 24 h and then stimulated with SeV for the indicated time points. The nuclear extracts and cytosolic extracts were isolated and subjected to Western blotting with abs against STAT1, Lamin B1, and β-Actin, respectively. (**C**) 293T cells were transfected with miR-200b-3p mimics, miR-200b-3p inhibitors, or their control oligonucleotides for 36 h. Levels of p-STAT1, STAT1, and ISG15 were determined by Western blot. Control oligonucleotides were used as a transfection control in all experiments. (**D, E**) 293T cells were co-transfected with different doses of pre-miR-200b-3p expression vector with WT (**D**) or mutant 3′ UTR TBK1 (**E**) for 36 h. The levels of ISG15, β-Actin, and TBK1 in the whole-cell lysates were detected by Western blot. (**F**) 293T cells were transfected with miR-200b-3p mimics or miR-200b-3p inhibitors, and induced by SeV infection. ELISA was performed to detect the concentration of IFN-β in the supernatants. Student’s t-test was used for statistical analysis of comparisons between groups. Bar graph shows the means ± SD, n = 3. Western blot data are representative of at least three independent experiments. SeV was used at a final concentration of 100 hemagglutinin units per ml.

Previous studies have shown that TBK1 can activate both NF-κB and IRF3 signaling to induce IFN-I expression (3 - 5). IFN-I then leads to the activation of the JAK-STAT signaling cascade and induces the expression of many ISGs (50). Therefore, we investigated whether miR-200b-3p inhibits IFN-I production by targeting TBK1. The results of dual luciferase reporter assays showed that miR-200b-3p mimics could reduce IFN-β and ISRE reporter activity, while the inhibitors of miR-200b-3p improved the activity (Fig. 6A). We also observed that transfection with pre-miR-200b-3p expression plasmid inhibited IFN-β and ISRE reporter activity in a dose-dependent manner (Fig. S7A and B).

Next, we investigated whether the nuclear translocation and phosphorylation of STAT1 were regulated by miR-200b-3p. We found that SeV-induced nuclear translocation of STAT1 was inhibited by miR-200b-3p (Fig. 6B). Consistently, miR-200b-3p mimics attenuated phosphorylation of STAT1, while miR-200b-3p inhibitors increased the level of phosphorylation (Fig. 6C). Furthermore, the effects of miR-200b-3p on SeV-stimulated expression of ISG15, which is one of the most rapidly and strongly induced ISGs (51, 52), was evaluated by Western blot. As expected, miR-200b-3p expression significantly suppressed ISG15 protein levels, and the suppression of ISG15 could be reversed in the presence of miR-200b-3p inhibitors (Fig. 6C).

We then co-transfected pre-miR-200b-3p expression plasmid and TBK1 expression plasmid with WT or MUT 3′ UTR to further confirm that the regulation of ISG15 by miR-200b-3p was associated with TBK1. The results showed that pre-miR-200b-3p has a dose-dependent inhibitory effect on the protein levels of ISG15 in the presence of TBK1 with WT 3′ UTR (Fig. 6D). However, there is no change in the ISG15 protein level was observed when co-transfected with TBK1 with MUT 3′ UTR (Fig. 6E). To further investigate the function of miR-200b-3p in IFN response, SeV was used as a stimulus to treat cells, and then the protein level of IFN-β was measured by ELISA. Inhibition of miR-200b-3p significantly increased IFN-β production, whereas expression of miR-200b-3p inhibited IFN-β production (Fig. 6F). Taken together, these results demonstrate that miR-200b-3p negatively regulates IFN-I production.

### Inhibition of miR-200b-3p restricts IAV replication in cells and attenuates viral pathogenicity in mice

**Fig 7:**
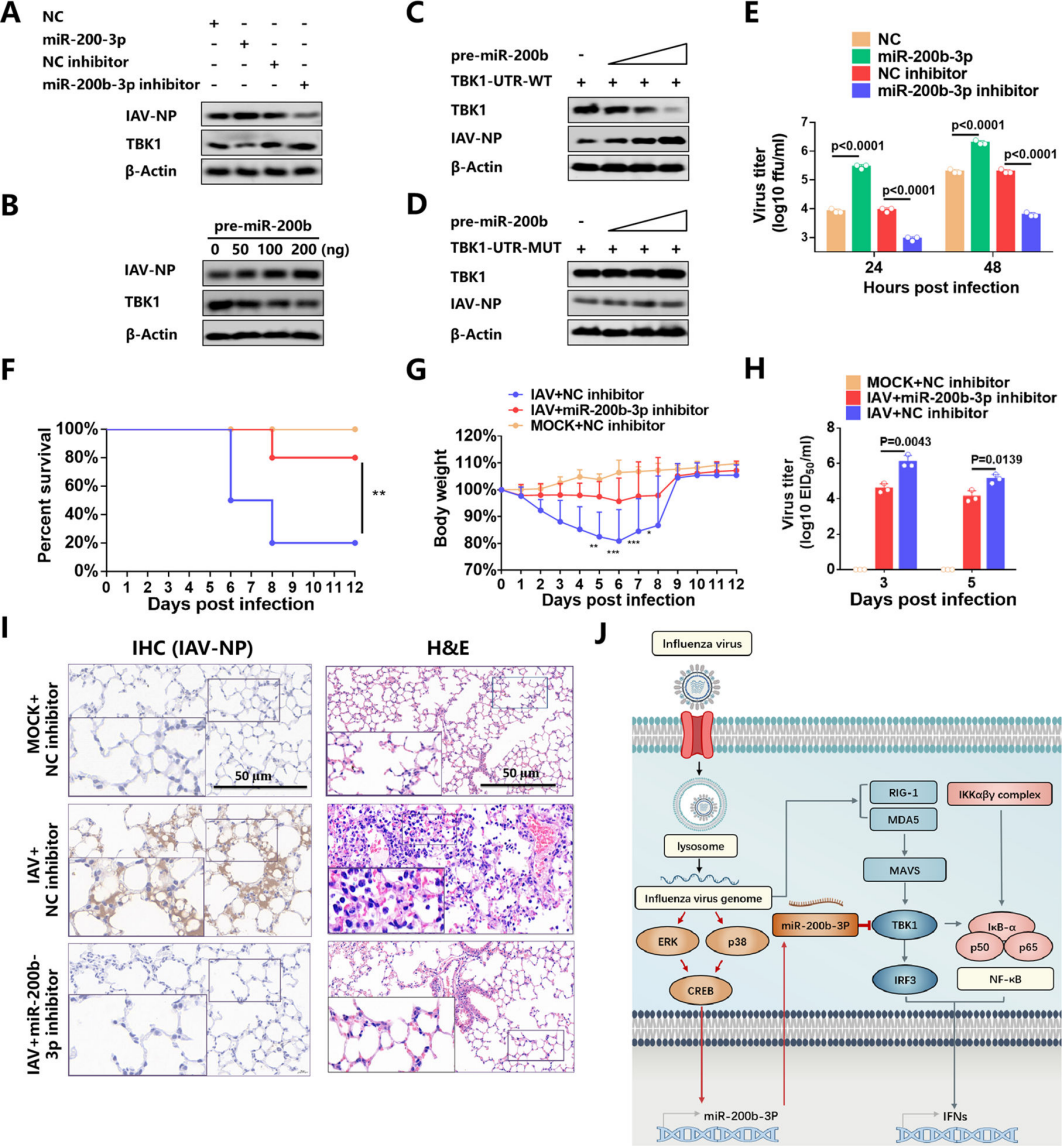
MiR-200b-3p inhibition improves the survival of IAV-infected mice. (**A**) 293T cells were transfected with miR-200b-3p mimics, miR-200b-3p inhibitors, or their control oligonucleotides. At 24 h after transfection, 293T cells were infected with IAV at 0.01 MOI. Western blots were performed with the indicated antibodies. (**B**) The different concentrations of pre-miR-200b-3p expression plasmid were transfected into 293T cells. At 24 h after transfection, cells were infected with IAV at 0.01 MOI for 24 h before Western blotting. (**C AND D**) 293T cells were co-transfected with pre-miR-200b-3p expression plasmid and TBK1 expression vector with WT 3′ UTR (**C**) or mutant 3′ UTR (**D**) for 24 h and then were infected with IAV at MOI of 0.01 for 24 h. Levels of TBK1, IAV-NP, and β-Actin in the whole-cell lysates were detected by Western blot. (**E**) 293T cells were transfected with NC, miR-200b-3p, NC inhibitor, or miR-200b-3p inhibitor for 24 h and infected with IAV at MOI 0.01. Culture supernatants were collected at different time points for TCID_50_ assay. (**F-J**) Mice were randomly divided into three groups: group 1, control group (MOCK + NC inhibitor); group 2, the IAV-infected and antagomir control-treated group (IAV + NC inhibitor); and group 3, the IAV-infected and antagomir-200b-3p-treated group (IAV + miR-200b-3p inhibitor). Mice in groups 2 and 3 were injected with 200 PFU IAV, while mice in group 1 were injected with an equal volume of DMEM. at 24 h post-viral infection, mice were administered with antagomir control or antagomir-200b-3p (60 mg/kg body weight, i.v.) on 2 consecutive days. Survival ratios (**F**) and body weight changes (**G**) were calculated (MOCK+NC inhibitor, n = 10; IAV+NC inhibitor, n = 10; and IAV+ miR-200b-3p inhibitor, n = 10). Error bars represent means ± SD. (**H**) Virus titers in the lungs of infected mice (n = 3) at 3 dpi (left) and 5 dpi (right). EID_50_: 50% infectious egg dose. (**I**) Virus loads at 5 dpi in the lungs of the above mentioned three groups of mice were determined by calculating the IAV-NP-positive cells by IHC, n = 3, scale bar = 50 µm. Pathological lesions in the lungs of mice infected with the indicated virus at 4 dpi were detected by hematoxylin and eosin (H&E) staining, n = 3, Scale bar = 50 µm. (**J**) Schematic diagram of the regulatory role of miR-200b-3p in the antiviral innate immune response. Body weight change was analyzed by two-way ANOVA test. The log-rank (Mantel-Cox) test was used to analyze the survival ratio. Student’s t-test was used for statistical analysis of comparisons between groups. Bar graph shows the means ± SD, n = 3. Western blot data are representative of at least three independent experiments.

Prevention and treatment of IAV infection are challenging due to its high mutational potential, numerous subtypes, and wide host range (53). As a key component of the innate immune system, rapid and robust induction of IFN-I plays a critical role in host defense against IAV infection (54). Since the enhancement of interferon by miR-200b-3p silencing has been demonstrated, we further evaluated the effect of miR-200b-3p inhibition on IAV replication and pathogenicity. The results showed that miR-200b-3p increased the level of IAV NP protein, whereas the miR-200b-3p inhibition decreased the NP protein expression (Fig. 7A). Furthermore, we observed that overexpression of pre-miR-200b-3p promoted NP protein level in a dose-dependent manner (Fig. 7B). In addition, we co-transfected pre-miR-200b-3p and TBK1 with WT or MUT 3′ UTR and then infected cells with IAV to confirm that the promotion of IAV replication by miR-200b-3p was related to its targeting effect on TBK1. The results showed that pre-miR-200b-3p increased NP protein expression in cells transfected with TBK1 carrying the WT 3′ UTR (Fig. 7C), and there was no significant effect on cells transfected with TBK1 carrying the MUT 3′ UTR (Fig. 7D). The IAV titers in the supernatant of cells transfected with miR-200b-3p mimics or miR-200b-3p inhibitors at each time point were then measured by virus titration, which showed that miR-200b-3p mimics significantly promoted IAV replication. Consistently, miR-200b-3p inhibitors had an inhibitory effect on replication (Fig. 7E). These results suggest that miR-200b-3p may promote IAV replication by inhibiting IFN-I production through the degradation of TBK1.

The therapeutic potential of miR-200b-3p inhibition was then evaluated in an IAV-infected mouse model. We injected miR-200b-3p inhibitor and NC inhibitor, respectively, into mice after infection with lethal doses of IAV and monitored animal survival ratio. The results showed that miR-200b-3p inhibition significantly attenuated virus pathogenicity. Injections of miR-200b-3p inhibitor after viral infection resulted in mild weight loss and 20% mortality. However, mice injected with NC inhibitor after viral infection showed severe disease and 80% mortality (Fig. 7F and G). Notably, viral titers in the lungs of the IAV+NC inhibitor group at 3 dpi were more than 30-fold higher than those detected in the IAV+miR-200b-3p inhibitor group (Fig. 7H). Meanwhile, weaker NP antigen signals were observed in the IAV+miR-200b-3p inhibitor group than in the IAV+NC inhibitor group (Fig. 7I). Also, the lungs of the IAV+NC-inhibitor group had moderate to severe bronchiolar necrosis and pulmonary edema, whereas these pathological changes were rarely observed in the IAV+miR-200b-3p inhibitor group (Fig. 7I). These results demonstrated that miR-200b-3p inhibition exerted a therapeutic role in IAV-infected mice. Taken together, we found that the CREB-mediated miR-200b-3p inhibits TBK1 and suppresses IFN-I production, through which the virus evades host’s innate immune response. Inhibition of miR-200b-3p can block this signaling cascade and restrict viral replication (Fig. 7J).

We also evaluated miR-200b-3p inhibitors *in vitro* and *in vivo* by using another well-known RNA virus, VSV, which is interferon-sensitive (39), to further confirm that miR-200b-3p inhibitors restrict viral replication by regulating interferon production. *In vitro*, the miR-200b-3p inhibitor significantly reduced VSV-G expression and virus titers, whereas miR-200b-3p mimics increased both of them (Fig. S8A and B). We also observed that pre-miR-200b-3p promoted VSV-G protein expression in a dose-dependent manner (Fig. S8C). *In vivo*, to silence endogenous miR-200b-3p, the chemically modified antisense oligonucleotide specific for miR-200b-3p was delivered to VSV-infected mice. All mice in the NC inhibitor group survived, while the mice in the VSV+NC inhibitor group had a lethality rate of 70%. In contrast, the VSV+miR-200b-3p inhibitor group showed a 60% reduction in lethality (Fig. S8D). In addition, the decrease in body weight was more pronounced in the VSV+NC inhibitor mice (Fig. S8E).

We also found that the VSV+NC inhibitor group contained higher viral titers than the VSV+miR-200b-3p inhibitor group (Fig. S8F). More VSV-G positive cells were consistently observed in the VSV+NC inhibitor group (Fig. S8G). Hematoxylin and eosin staining was then performed, which showed that vascular engorgement and hyperplasia were observed in brains from the VSV+NC inhibitor group. In contrast, no significant pathological lesions were observed in mouse brains from the VSV+miR-200b-3p inhibitor group (Fig. S8). In addition, the mRNA levels of IFN and ISGs, including IFN-β, Mx1, OAS1a, IFIT3, and ISG15, in mouse brains were also increased in the VSV+miR-200b-3p inhibitor group (Fig. S8G). Collectively, these data indicate that miR-200b-3p affects VSV pathogenicity *in vivo*, including viral replication level and mouse survivor ratio.

### MiR-200b-3p inhibitors suppress the replication of multiple pathogenic viruses

Viral infection of mammals induces the synthesis of type I IFN, which inhibits viral replication. The high susceptibility of type I IFN receptor knockout mice to infection by a variety of viruses provides strong evidence for the important role of the IFN system in protection against viral infection (55). We evaluated the effects of miR-200b-3p inhibitors on RABV (negative-stranded RNA virus), JEV (positive-stranded RNA virus), and HSV-1 (DNA virus), all of which pose a significant threat to public health worldwide.

**Fig 8:**
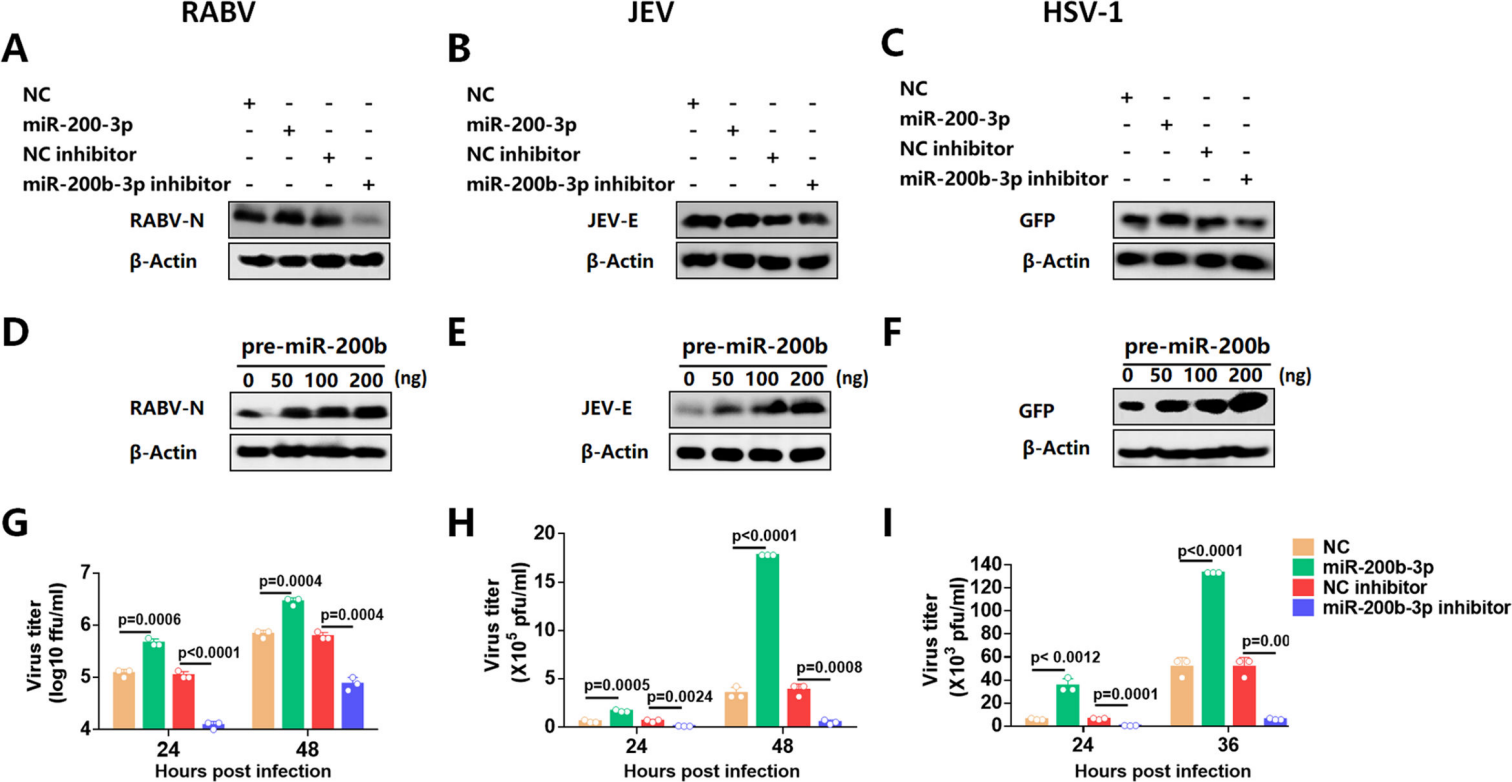
MiR-200b-3p expression promotes the replication of multiple RNA and DNA viruses. (**A-C**) N2a cells were transfected with NC, miR-200b-3p, NC inhibitor, or miR-200b-3p inhibitor At 24 h after transfection, 293T cells were separately infected with RABV at 0.01 MOI for 24 h (**A**), JEV at 0.01 MOI for 36 h (**B**), and HSV-1 at 0.01 MOI for 36 h (**C**) and then were harvested for Western blot. (**D-F**) N2a cells were transfected with the pre-miR-200b-3p plasmids. At 24 h after transfection, cells were infected with RABV at 0.01 MOI for 24 hr JEV at 0.01 MOI for 36 h, and HSV-1 at 0.01 MOI for 36 h. Viral protein levels were assessed by Western blot. The N2a cells were transfected with NC, miR-200b-3p, NC inhibitor, or miR-200b-3p inhibitor for 24 h and infected with RABV (**G**), JEV (**H**), and HSV-1-GFP (**I**) at 0.01 MOI, and then the culture supernatants were collected at different time points for TCID_50_ assay. Student’s t-test was used for statistical analysis of comparisons between groups. Bar graph shows the means ± SD, n = 3. Western blot data are representative of at least three independent experiments.

After transfection with miR-200b-3p mimics or miR-200b-3p inhibitor for 24 h, N2a cells were respectively infected with different viruses, and then the expression of different viral proteins was examined separately. The results showed that miR-200b-3p increased the levels of RABV N protein (Fig. 8A), JEV E protein (Fig. 8B), and GFP (recombinant HSV-1 carrying the GFP gene) (Fig. 8C). Consistently, miR-200b-3p inhibition decreased the expression of these proteins. In addition, we observed that pre-miR-200b-3p promoted the expression of these proteins in a dose-dependent manner (Fig. 8D to 8F). After transfection with miR-200b-3p mimics or miR-200b-3p inhibitors for 24 h, N2a cells were infected with RABV, JEV, and HSV-1, respectively, and the viral titers of cell supernatants were measured at different time points. The results showed that miR-200b-3p mimics significantly promoted viral replication, whereas miR-200b-3p inhibitors had an inhibitory effect on viral replication (Fig. 8G through I). Taken together, these results indicate that miR-200b-3p inhibitor can be used as a broad-spectrum inhibitor against viral infection.

## DISCUSSION

In this study, we identified miR-200b-3p as an IFN-I-related miRNAs, which inhibits the expression of TBK1 by directly binding with TBK1 3′ UTR. Upon viral infection, the upregulation of miR-200b-3p led to the reduction of TBK1-mediated IFN-I production and impaired the antiviral innate response. Importantly, miR-200b-3p inhibition has a broad-spectrum effect against viral replication by promoting IFN-I production both *in vitro* and *in vivo*. In addition, we found that activation of the transcription factor CREB by viral infection promotes the expression of miR-200b-3p. These data provided mechanistic insight into how miRNAs affect virus survival and immune escape.

Currently, the most effective ways to protect people from viral infections are vaccination and the use of antiviral drugs. However, viruses that pose an ongoing threat to humans, such as IAV, have the ability to frequently outcompete vaccines due to rapid evolution and the emergence of variants that are resistant to available antiviral drugs. Therefore, there remains an urgent need to develop effective treatments with broad-spectrum activity (56 - 58). IFN-I is effective against different groups of viruses through a variety of mechanisms (54). In addition, viruses can evade IFN inhibition by interfering with or disrupting molecules involved in IFN signaling (59). Here we identified a novel host factor involved in interferon regulation, miR-200b-3p.

Several studies showed that miRNAs regulate genes related to innate immune responses following viral infection. Previous studies suggested that enterovirus-induced miR-146a facilitates viral pathogenesis by suppressing IFN production by targeting IRAK1 and TRAF6 (60). Here, we further demonstrated that miR-200b-3p targets TBK1, through which miR-200b-3p inhibits TBK1-mediated NF-κB and IRF3 signaling. Interestingly, miR-429, which was found to inhibit the expression of IFN-β (Fig. 1B), also downregulates the TBK1 mRNA level (Fig. S9A). Since miR-429 also contains fragments complementary to the conservative sequence of TBK1 (CAGUAUU), it is possible that miR-429 inhibits the production of IFN-β by downregulating the level of TBK1, although the detailed mechanism needs further investigation.

The miR-200-3p mimics negatively affect the production of IFN-I and ISG, whereas the inhibition of miR-200b-3p promotes the production. The use of miR-200b-3p inhibitor significantly inhibited the proliferation of several viruses *in vitro*, including HSV-1, VSV, IAV, JEV, and RABV, and the introduction of mutations in the binding site of TBK1 3′ UTR with miR-200b-3p could restore this inhibition. MiRNAs are thought to target multiple mRNAs, called the targetome, to regulate gene expression. A single miRNA can regulate the protein synthesis of thousands of genes through direct or indirect effects (61). We may be far from discovering the last target of miR-200b-3p, and some potential targets may also be involved in regulating antiviral responses, which may trigger interesting future work.

Inhibition of microRNA is a very promising discovery in terms of reducing viral load *in vivo* and suggests a new therapeutic strategy to control viral infection (60). miR-122 inhibition by a specific inhibitor has been shown to restrict HCV infection and replication in chimpanzee models and in phase II clinical trials (62, 63). Thus, we introduced the miR-200b-3p inhibitor to demonstrate its potential application for viral treatment in the mouse model of IAV infection. Therapeutic administration at an early stage of viral infection effectively protected IAV-infected mice from virulent challenge. As previously described, this chemically modified miRNA inhibitor could cross the blood-brain barrier into the central nervous system via the intravenous route (64). The use of miR-200b-3p inhibitor significantly reduced mortality in a mouse model of VSV encephalitis, consistent with the important role of IFN in VSV pathogenecity. We further verified the relationship between miR-200b-3p, TBK1 and IFN production *in vivo*. In addition, the data provided new insights into how microRNA promotes viral survival and immune escape.

There is evidence that virus-induced host transcription factors induce miRNA expression by binding to their promoter regions (65). For example, the NF-κB p65 subunit binds to the promoter element of a subset of miRNAs genes and transcriptionally regulates their expression in response to LPS stimulation (66). In addition, miR-100, -146 a, and -150 were reported to be novel p53 and NF-κB p65/RelA responsive miRNAs (67). As a transcriptional regulator, CREB generally enhances the expression of target genes. Studies have shown that some noncoding CREB targets have been identified. CREB can directly bind to the regulatory sequences of miR-23a and increase miR-23a expression (68). CREB increases the expression of miR-373 by regulating its promoter (69). Here, we found that the promoter for miR-200b-3p contains a CREB binding site and that miR-200b-3p expression after IAV infection depends on CREB activation. NF-κB and MAPK pathway was activated upon IAV infection and played an important role in IAV-induced pathogenesis (70). Signal transduction pathways mediated by the MAPK family, including ERK1/2, JNK, and p38, contribute to the activation of transcription factors (71). Furthermore, ERK and p38 are potential upstream regulators in signaling CREB (72, 73), and CREB is also found to be activated in IAV infection (74). The transcription factors of microRNAs and their binding sites may differ or act synergistically under different stimulating factors, so the regulatory molecules of miR-200b-3p still need further investigation (20, 60, 75). In addition to the two RNA viruses, IAV and VSV, the DNA virus HSV-1 was also found to upregulate miR-200b-3p (Fig. S9B). Whether different types of viruses have the ability to regulate miR-200b-3p expression needs further research.

In conclusion, we have identified a novel miRNA-innate immunity interaction in which CREB-mediated miR-200b-3p leads to the degradation of TBK1 mRNA and suppression of IFN production, thereby allowing the virus to evade host immune attack. Inhibition of miR-200b-3p could block the IFN signaling cascade, thereby alleviating symptoms and mortality. Our findings highlight that miR-200b-3p is a potential drug target for a wide range of viral infections.

## SUPPLEMENTAL MATERIAL

Supplemental figures mbio.00867-23-s0001.doc Fig. S1 to S10.

ASM does not own the copyrights to Supplemental Material that may be linked to, or accessed through, an article. The authors have granted ASM a non-exclusive, world-wide license to publish the Supplemental Material files. Please contact the corresponding author directly for reuse.
