# Supplementary material for: Inhibition of miR-200b-3p confers broad-spectrum resistance to viral infection by targeting TBK1
Source: mBio. 2023 May 24;14(4):e00867-23. doi: 10.1128/mbio.00867-23 (PMC10470528; doi:10.1128/mbio.00867-23)
Supplement: Supplemental figures — Fig. S1 to S10. [file mbio.00867-23-s0001.doc]

**Supplementary Figures for**

**Inhibition of miR-200b-3p confers broad-spectrum resistance to viral infection by targeting TBK1**

An Fang^1,2#^, Yueming Yuan^1,2#^, Baokuen Sui^1,2^, Zhihui Wang^1,2^, Yuan Zhang^1,2^, Ming Zhou^1,2^, Huanchun Chen^1,2,3^, Zhen F. Fu^1,2^, Ling Zhao^*1,2,3^

^1^ State Key Laboratory of Agricultural Microbiology, Huazhong Agricultural University, Wuhan 430070, China

^2^ Key Laboratory of Preventive Veterinary Medicine of Hubei Province, College of Veterinary Medicine, Huazhong Agricultural University, Wuhan 430070, China

^3^ Hubei Hongshan Laboratory, Wuhan 430070, China

#An Fang and Yueming Yuan contributed equally to this work. The author’s order was determined alphabetically.

*Corresponding author: State Key Laboratory of Agricultural Microbiology, Huazhong Agricultural University, Wuhan, 430070, China. E-mail: [zling604@outlook.com](mailto:zling604@outlook.com), lingzhao@mail.hzau.edu.cn

**Supplemental Figures**

**
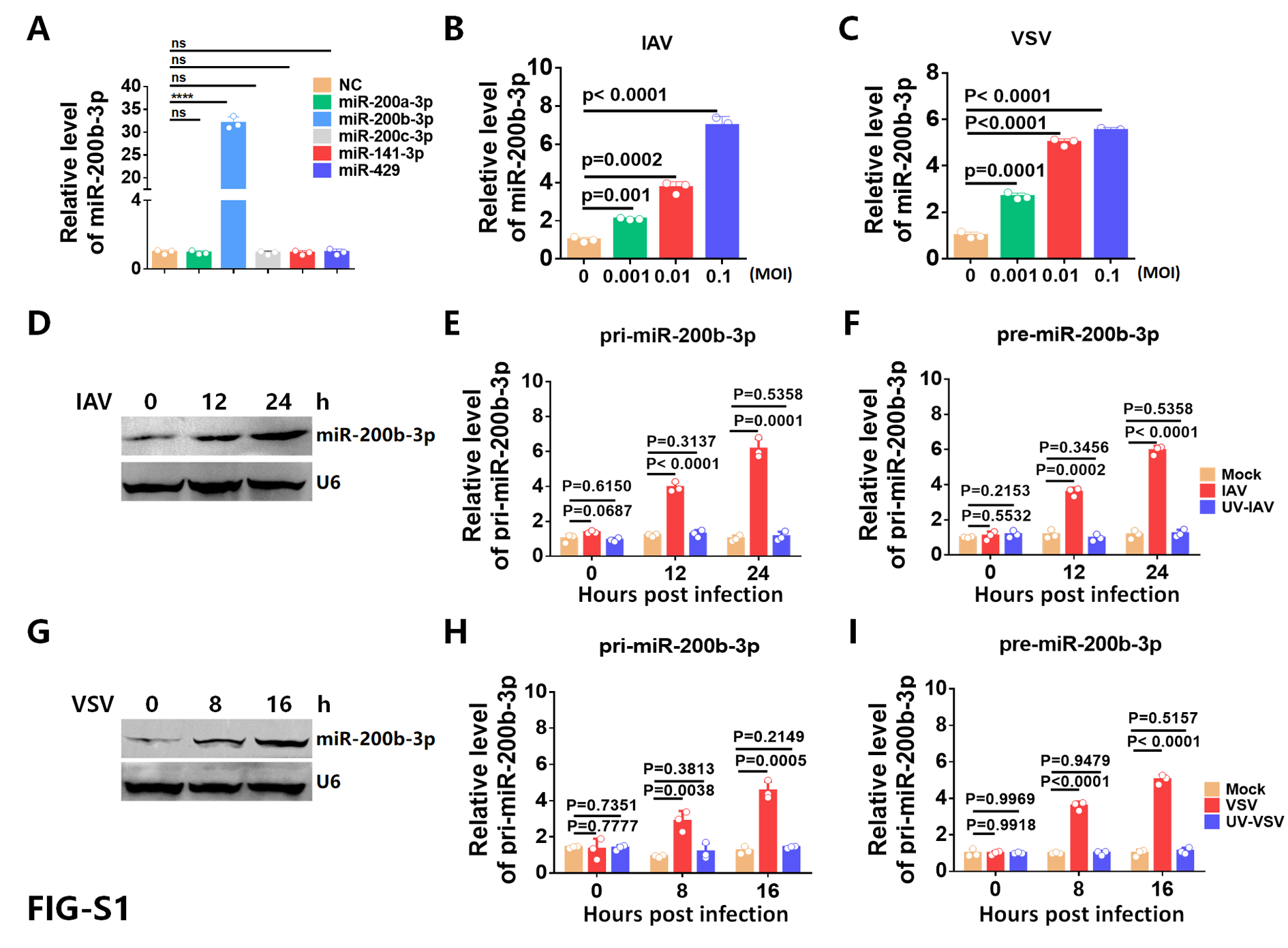
**

**FIG S1**

IAV and VSV infection upregulates the expression of miR-200b-3p. (A) 293T cells were transfected with microRNA-200a/200b/200c/141/429 mimics for 36 h. Primers specifically targeting miR-200b-3p were used for stem-loop qPCR analysis. 293T cells were infected with IAV at the 0.001, 0.01, 0.1 MOI for 16 h (B) or VSV (C) at the 0.001, 0.01, 0.1 MOI for 10 h. (D) The expression of miR-200b-3p in 293T cells infected with IAV at 0.01 MOI for the indicated times was quantified by Northern blot. (E, F) The levels of miR-200b-3p were detected by stem-loop qPCR. The expression of (E) Pri-miR-200b-3p and (F) Pre-miR-200b-3p in 293T cells infected with IAV at 0.01 MOI or incubated with UV-irradiated inactive IAV for indicated times was quantified by using qPCR analysis. (G) Expression of miR-200b-3p in 293T cells infected with VSV at 0.01 MOI for the indicated times was quantified by Northern blot. (H, I) 293T cells were infected with VSV at 0.01 MOI for the indicated times, or incubated with UV-irradiated inactive VSV for the indicated times. The qPCR was performed to detect the expression of (H) Pri-miR-200b-3p and (I) Pre-miR-200b-3p. Student’s t-test was used for statistical analysis of comparisons between groups. Bar graph shows the mean ± SD, n = 3.


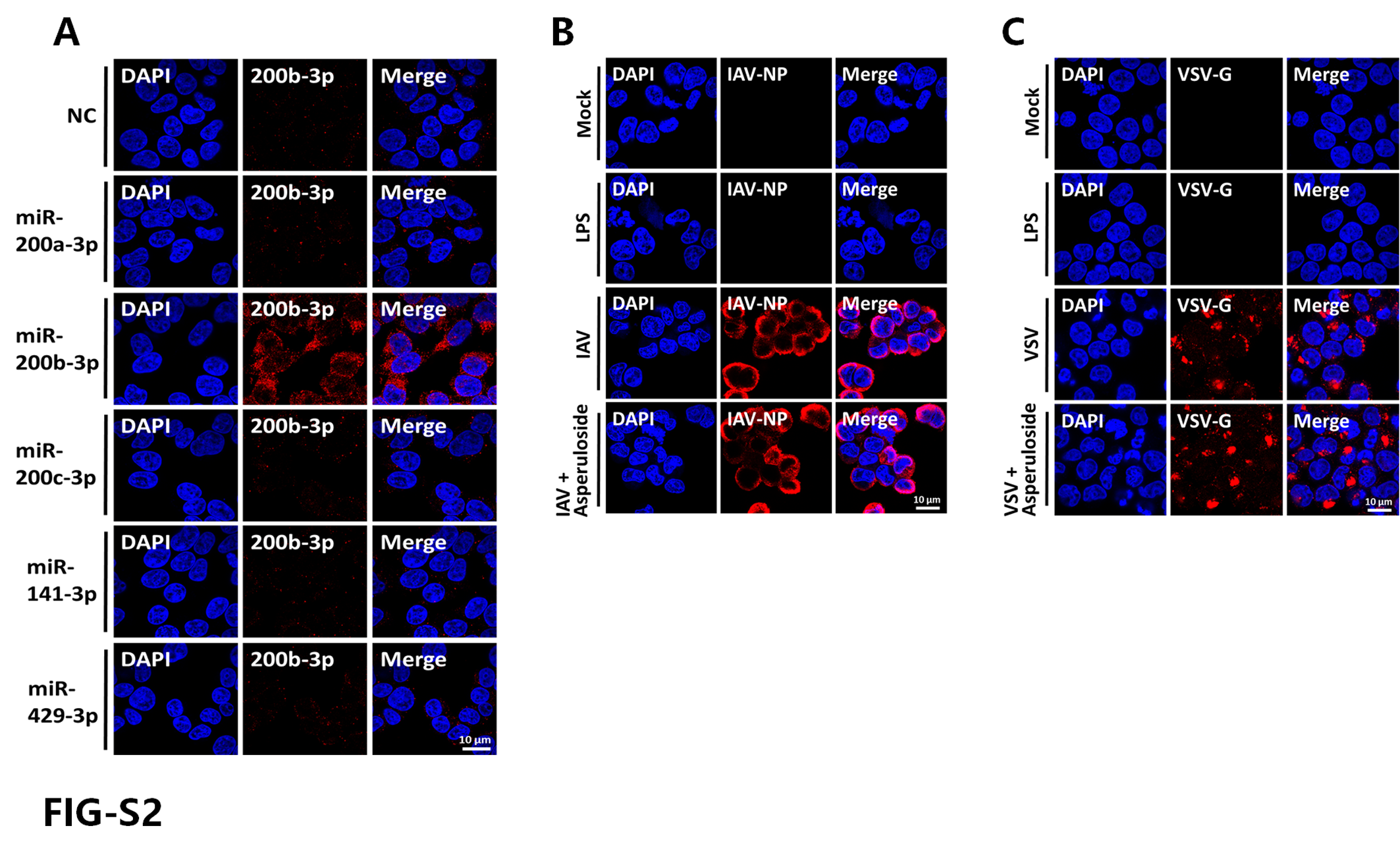


**FIG S2**

The specificity of the miR-200b-3p FISH assay. (A）293T cells were transfected with microRNA-200a/200b/200c/141/429 mimics for 36 h. Probes specifically targeting miR-200b-3p were used for FISH analysis. (B) Detection of IAV-NP protein in 293T cells infected with IAV at MOI of 0.01 for 24 h or treated with LPS (100 ng, 9 h) or infected with IAV at MOI of 0.01 and then treated with asperuloside (20 mM, 12 h) using FISH. (C) Detection of VSV-G protein in 293T cells infected with VSV at MOI of 0.01 for 16 h or treated with LPS (100 ng, 9 h) or infected with VSV and then treated with asperuloside (20 mM, 12 h) by FISH. Scale bar = 10 µm.


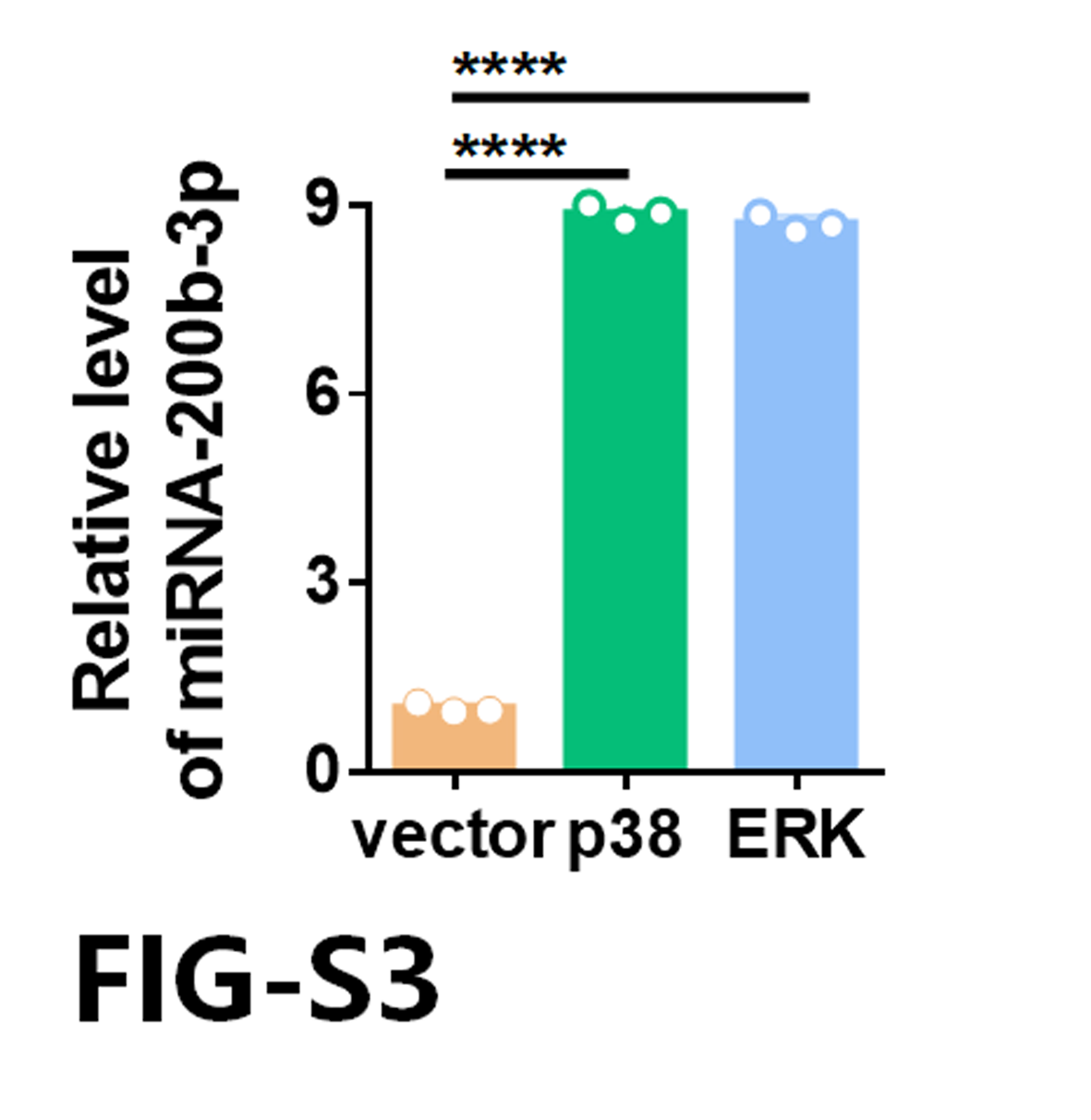


**FIG S3**

Activation of ERK and p38 signaling pathways upregulates the expression of miR-200b-3p. 293T cells were transfected with p38 or ERK expression plasmids for 36 h, and the expression of miR-200b-3p was detected by stem-loop qPCR analysis. Student’s t-test was used for statistical analysis of comparisons between groups. Bar graph shows the mean ± SD, n = 3.

**
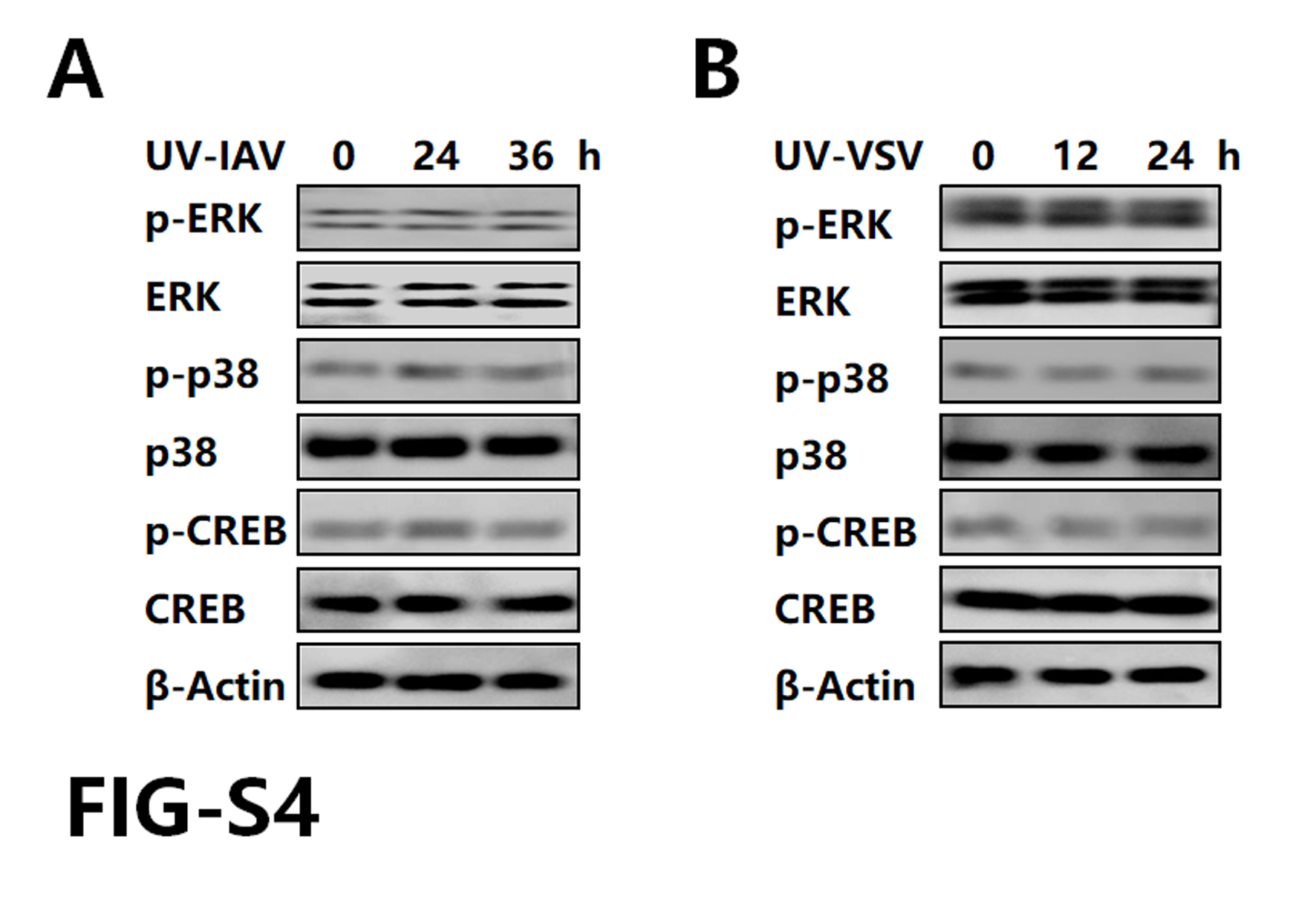
**

**FIG S4**

Inactivated IAV and VSV have no effect on p38/ERK-CREB pathway activation. (A and B) 293T cells were incubated with UV-IAV (A) or UV-VSV (B) for the indicated time points. Western blot was used to confirm the expression and phosphorylation of ERK, p38 and CREB.


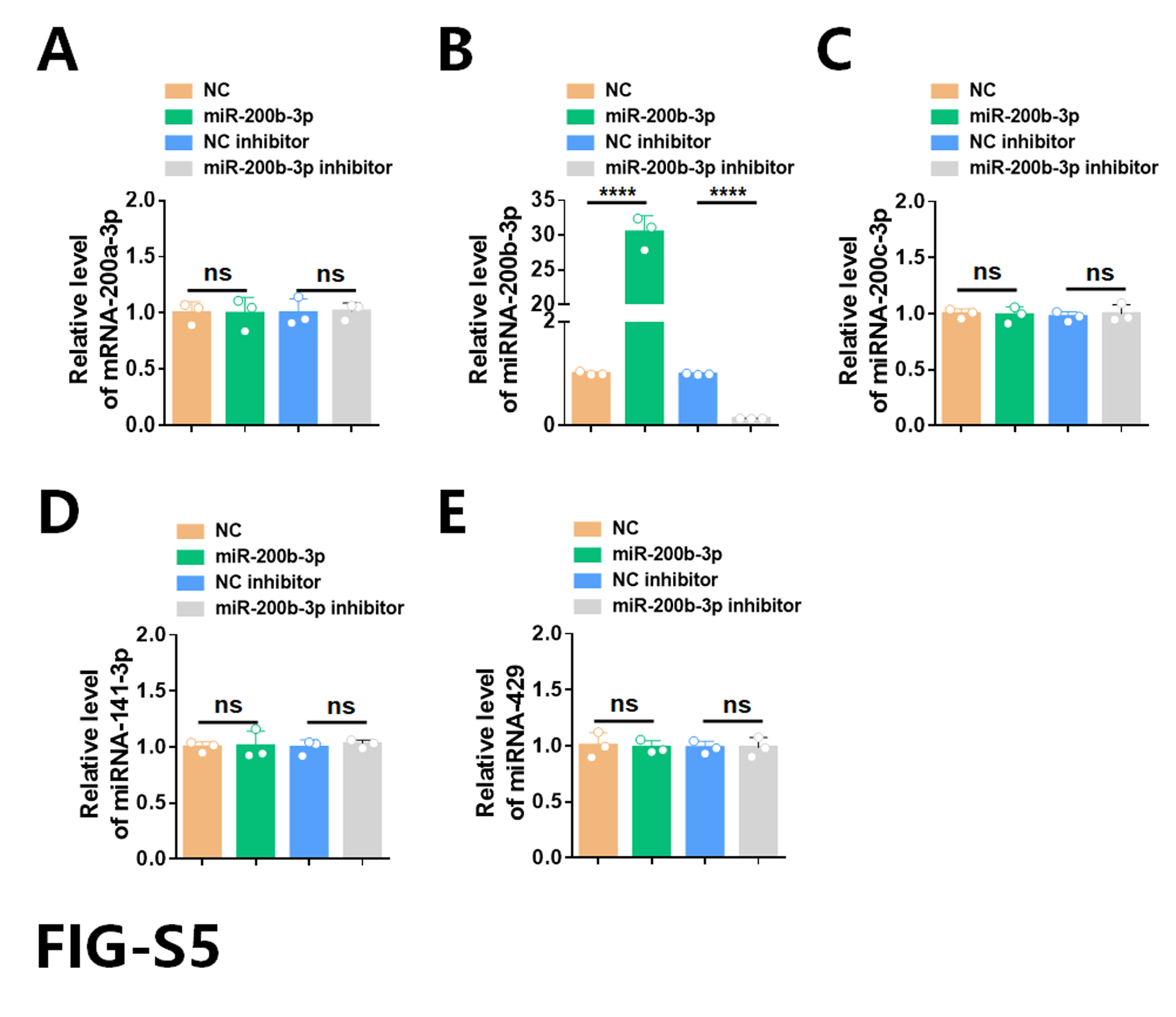


**FIG S5**

Target specificity of miR-200b-3p mimics and inhibitors. (A to E) 293T cells were transfected with NC mimics, miR-200b-3p mimics, NC inhibitors, and miR-200b-3p inhibitors for 36 h. Expression of miR-200a-3p (A), miR-200b-3p (B), miR-200c-3p (C), miR-141-3p (D), miR-429 (E) was detected by stem-loop qPCR assay. Student’s t-test was used for statistical analysis of comparisons between groups. Bar graph shows the mean ± SD, n = 3.

**
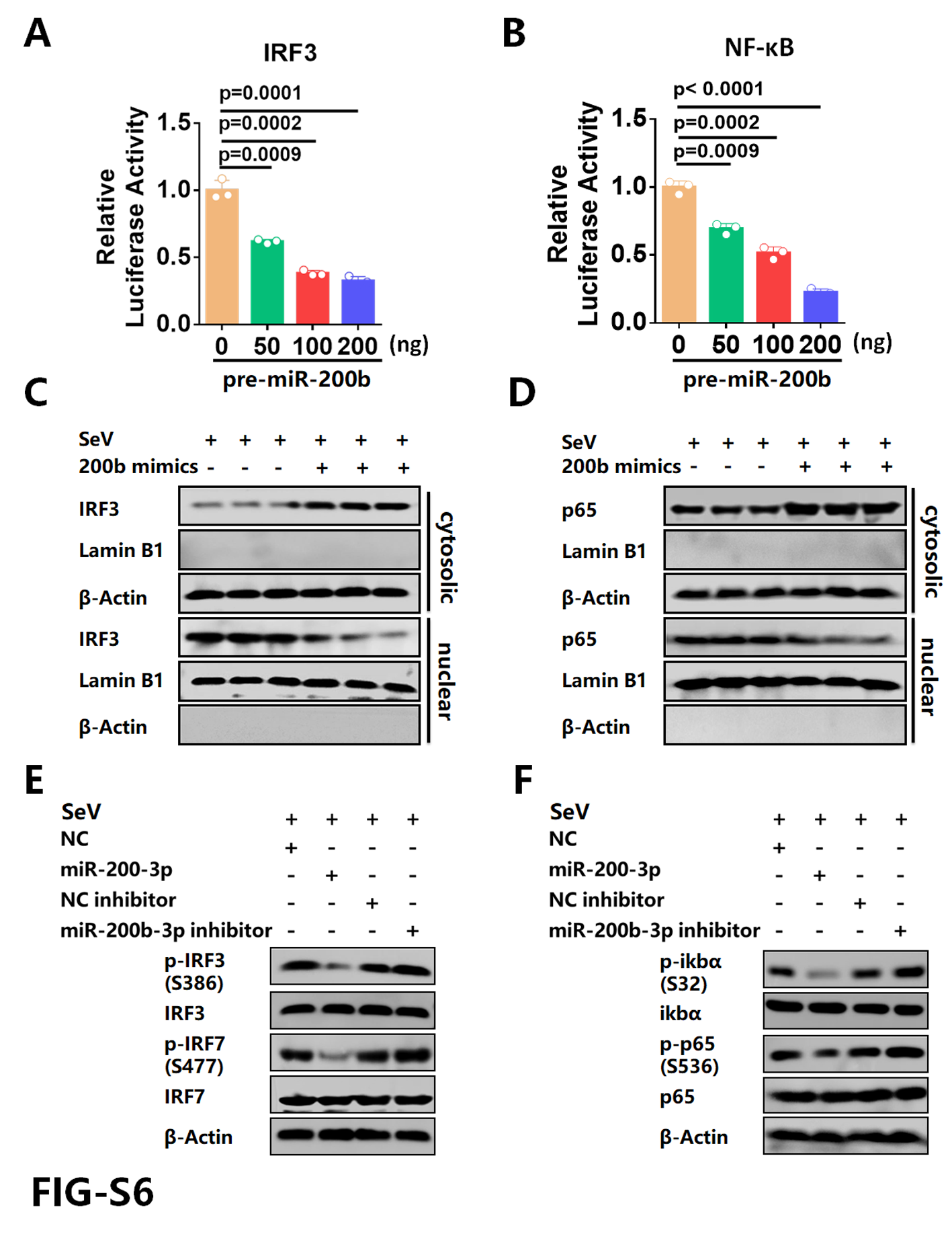
**

**FIG S6**

Regulation of IRF3 and NF-κB signaling pathways by miR-200b-3p. (A and B) Luciferase reporter plasmids (IRF3-Luc or NF-κB-Luc) and the pRL-TK plasmid were co-transfected into 293T cells with different concentrations of pre-miR-200b-3p expression plasmid. Cells were left uninfected or were infected with SeV for 12 h before reporter assays. (C and D) 293T cells were transfected with miR-200b-3p mimics or miR-200b-3p inhibitors for 24 h and then infected with SeV for 12 h. The nuclear and cytosolic extracts were isolated and subjected to Western blotting with Abs against IRF3, p65, Lamin B1, and β-Actin. (E and F) 293T cells were transfected with miR-200b-3p mimics, miR-200b-3p inhibitors, or their control oligonucleotides for 36 h. Phosphorylated and basic protein levels of the indicated proteins were determined. Control oligonucleotides were used as a transfection control in all experiments. Student’s t-test was used for statistical analysis of comparisons between groups. Bar graph shows the mean ± SD, n = 3. SeV was used at a final concentration of 100 hemagglutinin units per ml.


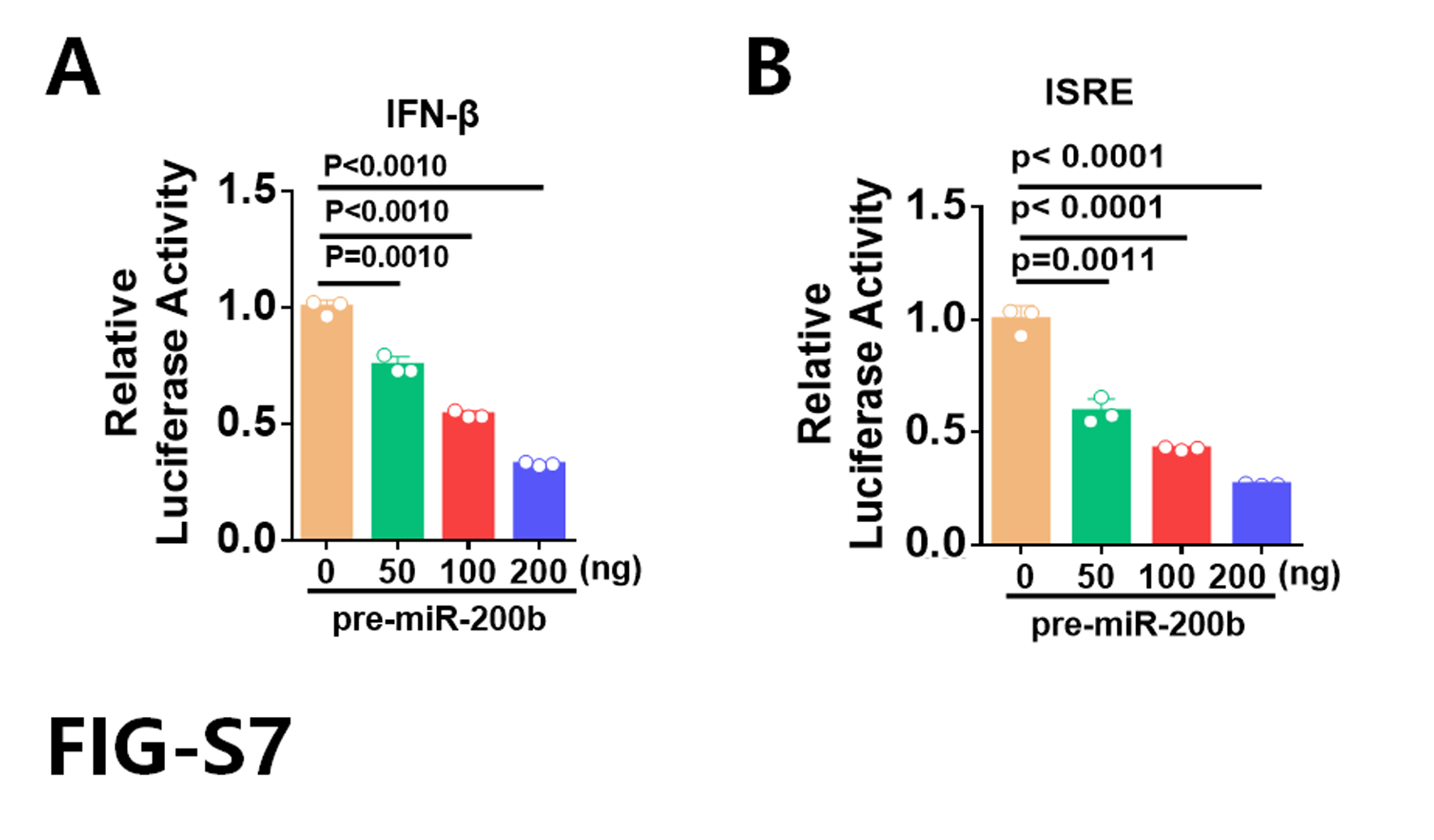


**FIG S7**

MiR-200b-3p negatively regulates the IFN-I signaling pathway. (A and B) Luciferase reporter plasmids (IFN-β-Luc or ISRE-Luc) and the pRL-TK plasmid were co-transfected into 293T cells, along with different concentrations of pre-miR-200b-3p expression plasmid. Cells were left uninfected or infected with SeV for 12 h at 24 h post-transfection prior to reporter assays. Student’s t-test was used for statistical analysis of comparisons between groups. Bar graph shows the mean ± SD, n = 3. SeV was used at a final concentration of 100 hemagglutinin units per ml.


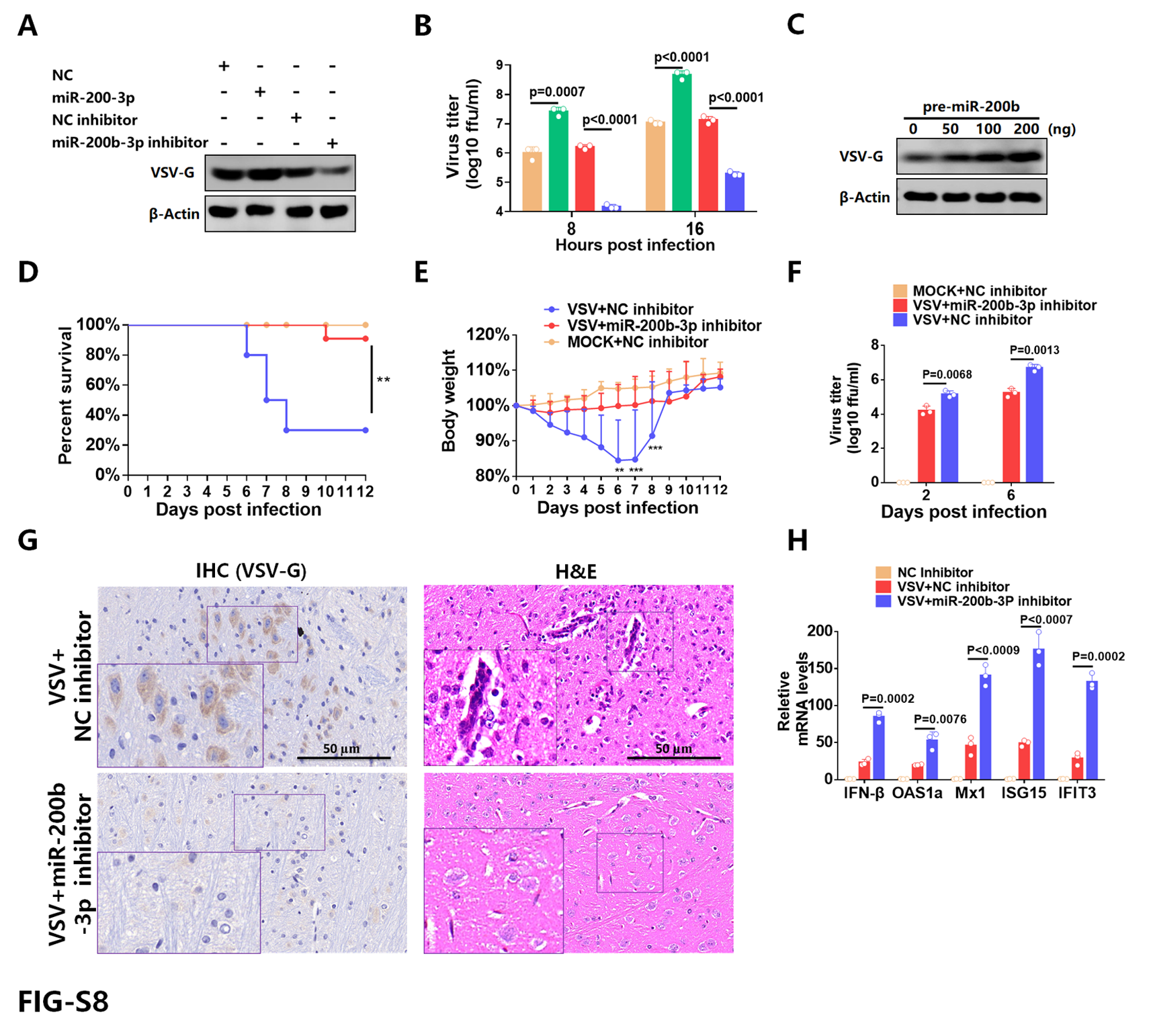


**FIG S8**

MiR-200b-3p inhibition improves the survival of VSV-infected mice. (A) Cells were separately transfected with NC, miR-200b-3p, NC inhibitor, or miR-200b-3p inhibitor and infected with VSV at MOI 0.01 for 16 h and then were harvested for Western blot. (B) 293T cells were transfected with NC, miR-200b-3p, NC inhibitor, or miR-200b-3p inhibitor for 24h and infected with VSV at MOI 0.01. Cultural supernatants were acquired at time points for TCID_50_ assay. (C) 293T cells were transfected with the pre-miR-200b-3p plasmids. 293T cells were infected with VSV at 0.01 MOI for 16 h. VSV-G protein levels were assessed by Western blot. (D-G) Mice were treated with antagomir-200b-3p (miR-200b-3p inhibitor (60 mg/kg body weight, i.v.)) or antagomir-NC (NC inhibitor)) after VSV infection (10^7^ FFU, i.n.), or antagomir-NC (NC inhibitor) after DMEM injection. The survival ratios (D) and body weight changes (E) were calculated (MOCK + NC inhibitor, n = 10; VSV + miR-200b-3p inhibitor, n = 10; VSV + NC inhibitor, n = 10). (F) The VSV titers in the whole brain at 2 and 6 dpi. (G) Viral loads at 5 dpi in mouse brains of the above mentioned three groups of mice were determined by detecting the VSV-G positive cells by IHC, n = 3, scale bar = 50 µm. (G) Pathological lesions in the brain of mice infected with VSV at 5 dpi were detected by hematoxylin and eosin (H&E) staining. n = 3, scale bar = 50 µm. (H) Transcription levels of indicated IFN-related genes were analyzed by qPCR. Body weight change was analyzed by two-way ANOVA test. The log-rank (Mantel-Cox) test was used to analyze the survival ratio. Student’s t-test was used for statistical analysis of comparisons between groups. Bar graph shows the mean ± SD, n = 3. Western blot data are representative of at least three independent experiments.

**
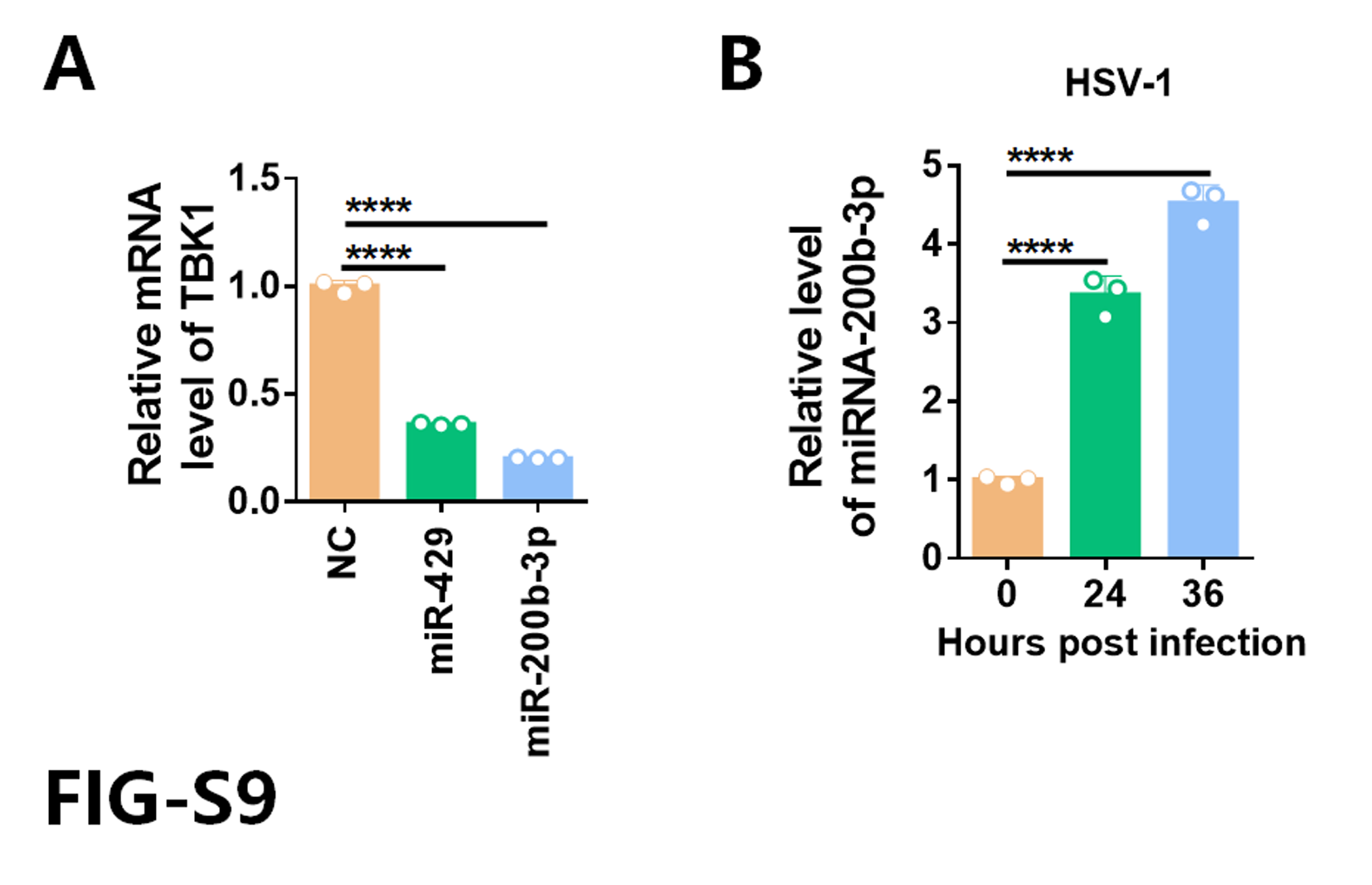
**

**FIG S9**

MiR-429 down-regulate TBK1 mRNA levels. HSV-1 infection up-regulate miR-200b-3P. (A) 293T cells were transfected with NC mimics, miR-429 mimics, or miR-200b-3p mimics, respectively, for 36 h. TBK1 mRNA levels were then measured by qPCR. (B) 293T cells were infected with HSV-1 at MOI 0.01 for 24 or 36 h. Stem-loop qPCR was performed to detect the expression of miR-200b-3p. Student’s t-test was used for statistical analysis of comparisons between groups. Bar graph shows the mean ± SD, n = 3.

**
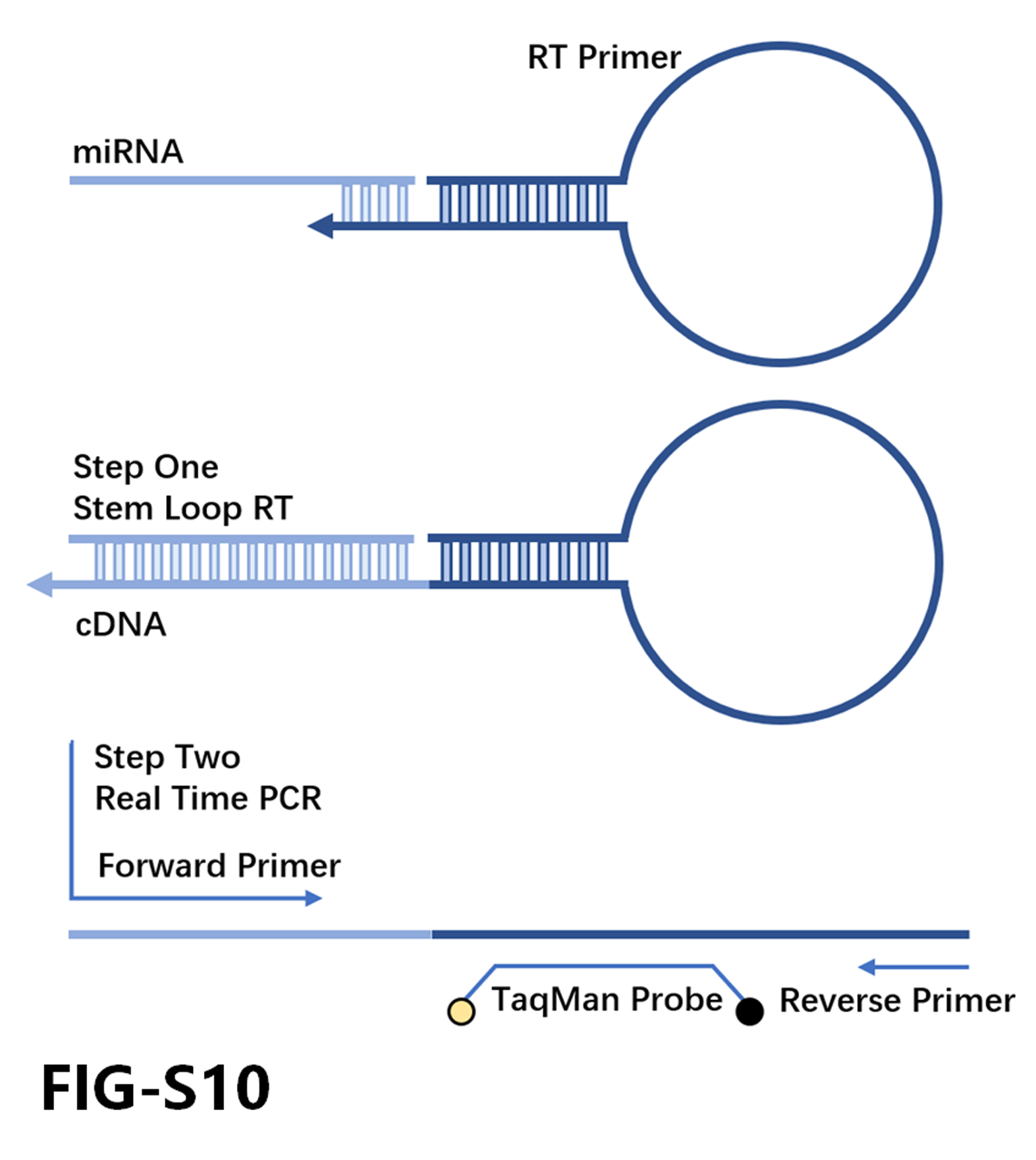
**

**FIG S10**

Schematic diagram illustrating the principle of stem-loop primer-based microRNA detection (stem-loop qPCR). Stem-loop RT primers bind to at the 3′ portion of miRNA molecules and are reverse transcribed with reverse transcriptase. The RT product is then quantified by using TaqMan qPCR with miRNA-specific forward primer, reverse primer and TaqMan probe.
